# RNASeq analysis of drought-stressed guayule reveals the role of gene transcription for modulating rubber, resin, and carbohydrate synthesis

**DOI:** 10.1038/s41598-021-01026-7

**Published:** 2021-11-03

**Authors:** Chen Dong, Grisel Ponciano, Naxin Huo, Yong Gu, Daniel Ilut, Colleen McMahan

**Affiliations:** 1 USDA Agricultural Research Service, Western Regional Research Center, Albany, CA 94710 USA; 2 Plant Breeding and Genetics Section, School of Integrative Plant Science, Cornell University, Ithaca, NY 14853 USA

**Keywords:** Biotechnology, Computational biology and bioinformatics, Molecular biology, Plant sciences

## Abstract

The drought-adapted shrub guayule (*Parthenium argentatum*) produces rubber, a natural product of major commercial importance, and two co-products with potential industrial use: terpene resin and the carbohydrate fructan. The rubber content of guayule plants subjected to water stress is higher compared to that of well-irrigated plants, a fact consistently reported in guayule field evaluations. To better understand how drought influences rubber biosynthesis at the molecular level, a comprehensive transcriptome database was built from drought-stressed guayule stem tissues using de novo RNA-seq and genome-guided assembly, followed by annotation and expression analysis. Despite having higher rubber content, most rubber biosynthesis related genes were down-regulated in drought-stressed guayule, compared to well-irrigated plants, suggesting post-transcriptional effects may regulate drought-induced rubber accumulation. On the other hand, terpene resin biosynthesis genes were unevenly affected by water stress, implying unique environmental influences over transcriptional control of different terpene compounds or classes. Finally, drought induced expression of fructan catabolism genes in guayule and significantly suppressed these fructan biosynthesis genes. It appears then, that in guayule cultivation, irrigation levels might be calibrated in such a regime to enable tunable accumulation of rubber, resin and fructan.

## Introduction

Guayule (*Parthenium argentatum* A. Gray), the perennial desert shrub native to the Southwestern United States and northern Mexico, is a rubber-producing plant adapted to arid and semiarid regions^1,2^. Large scale guayule cultivation in the United States could provide a domestic source of natural rubber (NR, *cis*-1,4-polyisoprene) necessary for national defense, modern transportation, and medicine^3,4^. One challenge in guayule crop management is to maximize rubber yield while maintaining efficient irrigation water use. Techno-economic models for guayule cultivation point to water use as a major cost to growers^5^. However, high yields of rubber can be achieved with subsurface drip irrigation^6^. In that study, as in others^7–11^, guayule biomass and rubber yield responded positively to total water applied; however plant rubber content (wt%) consistently increased as irrigation water levels decreased. The possibility that drought stress may positively impact rubber biosynthesis is compelling, especially since water availability is an economic and environmental constraint in the Southwestern US.

In addition to NR, guayule produces other secondary metabolites in appreciable amounts such as resin (5–12% dry weight^12^). Guayule resin constitutes a mixture of terpenoids, sterols, fatty acids, aromatic compounds, and low-molecular weight rubber, but the precise composition is much more complex^13^. Among the most abundant resin extractable compounds are argentatins (20–30%) which may have anti-cancer properties^14^ and the guayulins (10–15%) known to have fungistatic and miticide activities^15^. The biological roles of individual resin compounds have likely evolved in response to biotic and abiotic stresses, and/or have ecological functions as is the case of many plant secondary metabolites^16^. Interestingly, the cellular locations of resin and NR production in guayule are in close proximity. The terpene components of resin are produced in resin canals in the bark parenchyma tissue and in the pith of stems, and NR is predominantly found in a single layer of epithelial cells surrounding the resin canal^17^. This intimate spatial distribution of the two metabolites makes it difficult to extract resin-free rubber (residual resin in the rubber extract compromises its stability^18^), and vice-versa. Proposed commercial uses of guayule resin include in wood preservatives, paints and adhesives^19^, asphalt^20^, and pharmaceutical compounds^14,21^.

Another abundant metabolite produced by guayule is the carbohydrate fructan (up to ~ 10% dry weight^22^), a biobased chemical feedstock. Fructans are fructose-based polymers with the predominant role of serving as carbon reserves when metabolic demand exceeds carbon availability^23^, but are also considered to be protective agents against abiotic stresses^24–26^. In guayule, fructans accumulate mainly in the root and stems and to lesser extent in leaves^22^. Fructan levels fluctuate seasonally^22,27^, and are highest at the onset of and during winter conditions parallel to rubber biosynthesis^28^.

The precise mechanism of in vivo NR synthesis remains to be elucidated, but in general the biosynthetic pathway comprises two main phases. First, the monomeric unit isopentenyl pyrophosphate (IPP), the rubber polymer-monomer, and the allylic initiator (typically farnesyl pyrophosphate, FPP) required for rubber biosynthesis, are produced by the cytosolic mevalonate pathway (MVA). Importantly, the IPP and FPP pools are substrates for biosynthesis of all downstream isoprenoids (terpenes, dolichols, sterols, etc.) many of which make up guayule resin. The second phase involves the actual NR synthesis by an enzymatic complex (rubber transferase, RuT) of unknown identity. The RuT is presumed to be localized on the surface of a vesicle derived from the endoplasmic reticulum and known as a rubber particle (RP). Current models propose FPP first binds to the RuT-binding site, and subsequently thousands of IPP undergo condensation reactions to produce the rubber molecule. As NR is synthesized it accumulates inside the RP while various distinctive proteins associate to stabilize it and/or modulate rubber biosynthesis^29–32^.

It is well established that cold stress elicits rubber biosynthesis in guayule stem bark tissues^33–35^, but water stress has also been reported to have a positive effect in guayule rubber content^6,11,36^. With the goal of identifying molecular players of drought-stressed guayule impacting rubber biosynthesis, we analyzed the transcriptome of field-grown plants under two irrigation water treatments: drought-like and fully irrigated control. A comprehensive transcriptome database was built using genome-guided^37^ and de novo RNA-seq assembly. Our comparative analyses of the global transcription expression revealed that transcripts related to rubber biosynthesis were mostly down-regulated. Further, transcripts related to fructan biosynthesis (but not fructan catabolism) were also down-regulated. Expression regulation of genes encoding enzymes responsible for terpene resin biosynthesis was mixed.

## Results and discussion

### Transcriptome sequencing and read assembly

To elucidate the molecular responses impacting secondary metabolite production in guayule during drought stress, six libraries from drought-treated and control stem tissue RNA were sequenced using Illumina HiSeq 2000 platform. In total, 196,861,972 raw pair-end reads with a read length of 2 × 150 bp were generated (Table 1). After quality trimming, 97.11% of cleaned reads were recovered and used for sequence assembly. A comprehensive transcriptome database was built using both genome-guided and Trinity de novo RNA-Seq assembly methods incorporated in the PASA pipeline^38^. A total of 229,190 unique contigs were obtained with non-redundant cut off at 95%, following a further filtration process to retain transcripts longer than 300 bp only. The most highly expressed transcripts that represent 84% of the total normalized expression data achieved an N50 of 1851 bps. The total accumulated size of the assembled transcripts was approximately 249 Mb, with length ranging from 300 to 17,875 bp. Additionally, 90.75% of all clean reads perfectly mapped back to the reference transcriptome, suggesting a strong representation of the read input and the quality of the assembly was sufficient for downstream analysis.

**Table 1:** Transcriptome assembly summary statistics. Annotation carried out with BlastX and cut-off at 1E-5.

**Assembly statistics**		
Number of unigenes	229,190	
GC content	40.41%	
N50 (bp)	1,640	
Media contig (bp)	869	
Average contig (bp)	1,172	

Database	Contig counts	% annotated
**Functional annotation**		
Nr	143,286	62.52
Swiss-prot	111,873	48.81
Trembl	142,449	62.15
PlantTFDB	2,759	1.20
KEGG	65,879	28.74
GO	34,502	15.37
Total Transcripts	229,190	

### Differential gene expression and functional enrichment

**Figure 1:**
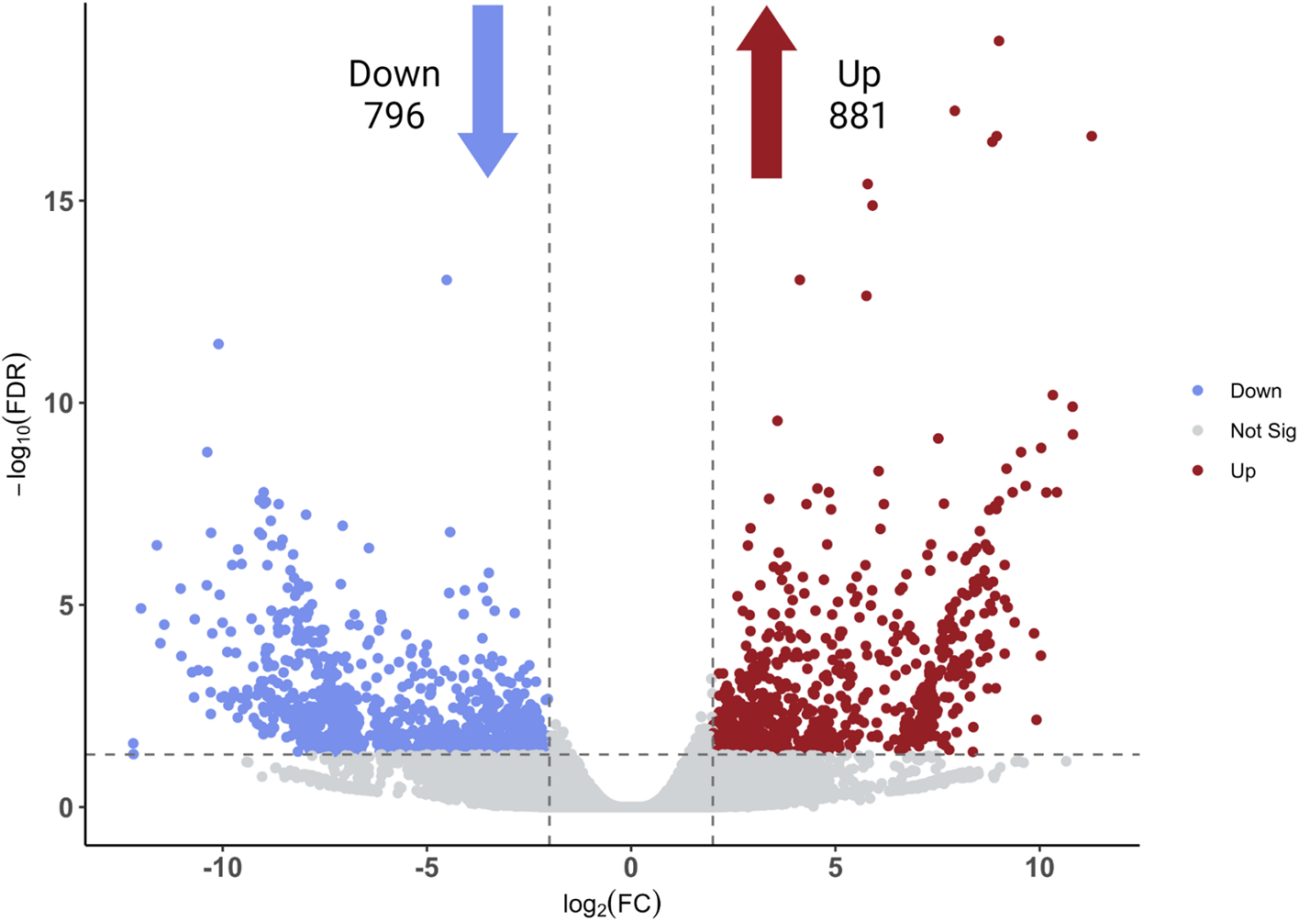
Volcano plot representation of differential expression. Red and blue points mark the transcripts with significantly increased or decreased expression in drought stressed plants versus control plants, respectively (FDR < 0.05). The x-axis shows log2 fold-changes in expression and the y-axis log10 false discovery rate of a transcript being differentially expressed.

To quantify abundances of each unigene, Kallisto^39^ was used to generate TPM (transcripts per million), reflecting the relative molar concentration of transcripts in each sample. edgeR^40^ was deployed to identify the differentially expressed transcripts with FDR < 0.05 and log2 fold change > 2. As a result, 1677 were found to be significantly differentially expressed, with 881 up-regulated and 796 down-regulated transcripts in drought-stressed tissue compared to the control (Fig. 1).

To predict and analyze the function of the differentially expressed unigenes, we assessed the assembled transcripts using BLASTX search against databases listed in Table 1. Among the 229,190 transcripts, 143,286 (62.52%) could be annotated in Nr (NCBI non-redundant protein sequences), 51,596 (47.94%) in Pfam (protein family database), 104,111 (45.43%) in Swiss-Prot (a manually annotated and reviewed protein sequences database), 34,502 (15.37%) in GO (Gene Ontology), and 40,917 (17.85%) in KEGG (Kyoto Encyclopedia of Genes and Genomes). Overall, 70.15% transcripts were significantly matched to known genes in the public databases mentioned above (Table 1), while those with no significant protein matches may represent novel proteins and long non-coding RNAs in guayule.

Transcription factors (TFs) regulate the transcription of genes and play key regulatory roles in plant growth, development, and response to environmental stress. Our analysis revealed that 2759 transcripts (1.20%) encode putative TFs that can be classified into 56 families (Supplementary Table S1). Most abundantly represented were the basic helix-loop-helix (bHLH) family (292, 10.58%), followed by cysteine2-histidine2 zinc finger (C2H2) family (179, 6.49%), basic leucine zipper (bZIP) family (166, 6.02%), no apical meristem/ATAF1–2/cup-shaped cotyledon (NAC) family (160, 5.80%), myeloblastosis (MYB) and related (296, 10.73%) and the cysteine3histidine (C3H) family (139, 5.04%). It is worth noting that MYB-related, bHLH and WRKY families are known to regulate secondary metabolism pathways in plants. In the rubber producing tree *Hevea brasiliensis* (Hevea), HbMYC2 was found to be highly expressed in bark and possibly positively regulating the RP associated gene *HbSRPP*^41^ and to activate NR biosynthesis genes *HbFPS1* and *HbSRPP1*^42^.

The three most enriched differentially expressed TF families were constans-like (2 transcripts up- and 7 down-regulated), MYB-related (9 transcripts up), golden2-like (1 transcript up- and 5 down-regulated) and bHLH (3 up- and 1 down-regulated). More than 60% of CO-like TF family transcripts (9 of 15) were significantly enriched under drought condition, among which more than 77% were down-regulated. The identification of this large set of TFs, along with their expression profiling under drought stress, provides a rich resource for future characterization of specific roles of TFs in rubber biosynthesis pathway under drought stress condition. Interestingly, we performed a comparative analysis of the transcriptome profile from greenhouse grown guayule subjected to cold^43^, and our drought stressed guayule transcriptome found a total of 58 significantly differentially expressed contigs under both stresses; sixteen of these encode transcription factors (Supplementary Table S2).

**Figure 2:**
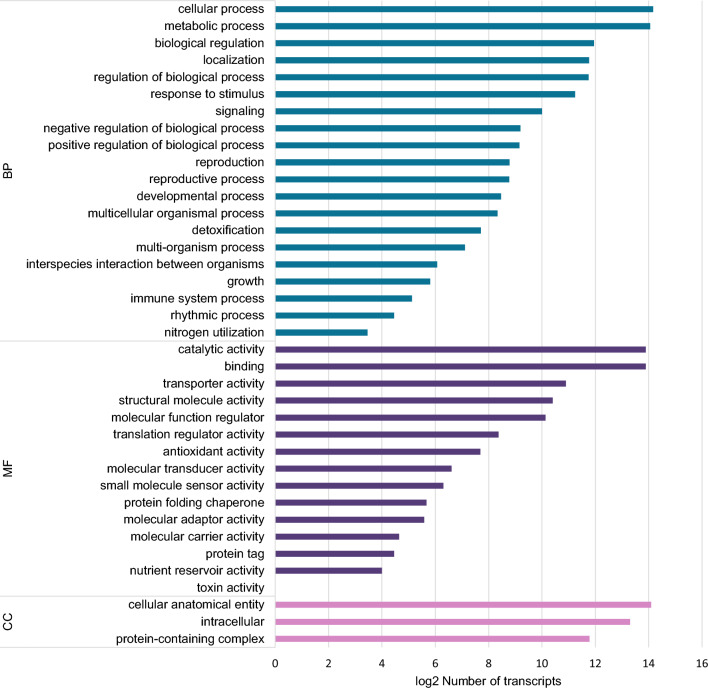
Gene Ontology (GO) analysis of RNA-seq. GO term classification of the entire transcriptome. *BP* biological process; *CC* cellular component; *MF* molecular function.

Gene Ontology assignments were used to determine the potential functions of the transcripts and classify them based on various biological processes. In total, 34,502 contigs were assigned to three major functional categories: biological process, molecular function, and cellular component (Fig. 2). The GO sub-categories with the largest transcripts were ‘cellular process’, ‘metabolic process’, ‘catalytic activity’, ‘binding’, and ‘cellular anatomical entity’.

For GO enrichment analysis, we took consideration of correlations within a gene set with modest variability in expression but which may exhibit a general trend. The entire contig list, ranked based on the level of fold change without eliminating the ones considered non-significant, was supplied into Kolmogorov–Smirnov test. Overall, a total of 33 ontology terms (Supplementary Table S3) were enriched with FDR < 0.01. Interestingly, only one set involving terpene synthase activity (GO:0010333) was recognized as down-regulated in the drought stressed plants. Seven out of the remaining 32 up-regulated gene sets were, not surprisingly, related to redox activity grouped across both biological process and molecular function. We also found 5 enrichment sets of transcripts associated with proton transport coupled with ATP production and decomposition via oxidative phosphorylation and the tricarboxylic acid cycle as well as transcription and translation machinery, indicating that drought stressed plants were in a highly active energetic state through cellular respiration. Notably, glycolytic process (GO:0006096) producing pyruvate as end-product, which after oxidative decarboxylation fuels the carbon source for rubber biosynthesis^44^, is also significantly enriched in drought-stressed plants.

**Figure 3:**
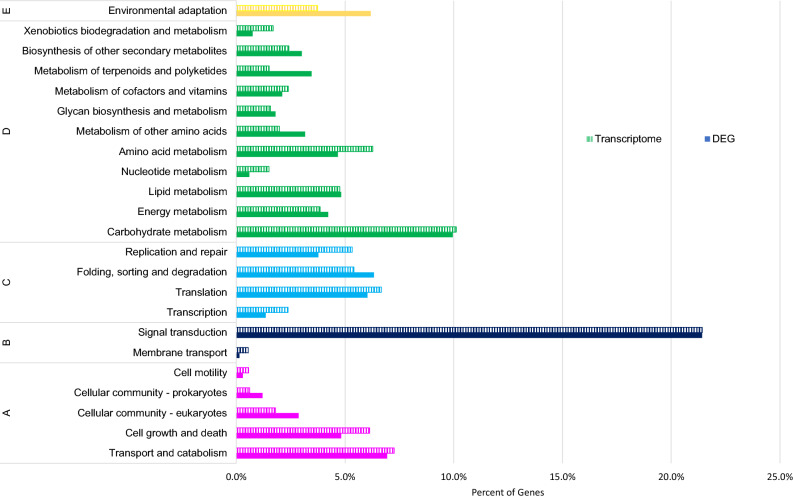
KEGG classification of unique sequences in both entire transcriptome (dashed line) and differentially expressed contigs (solid line). (A) Cellular processes; (B) Environmental information processing; (C) Genetic information processing; (D) Metabolism; and (E) Organismal systems. A total of 65,879 unique sequences were classified in the KEGG database.

**Figure 4:**
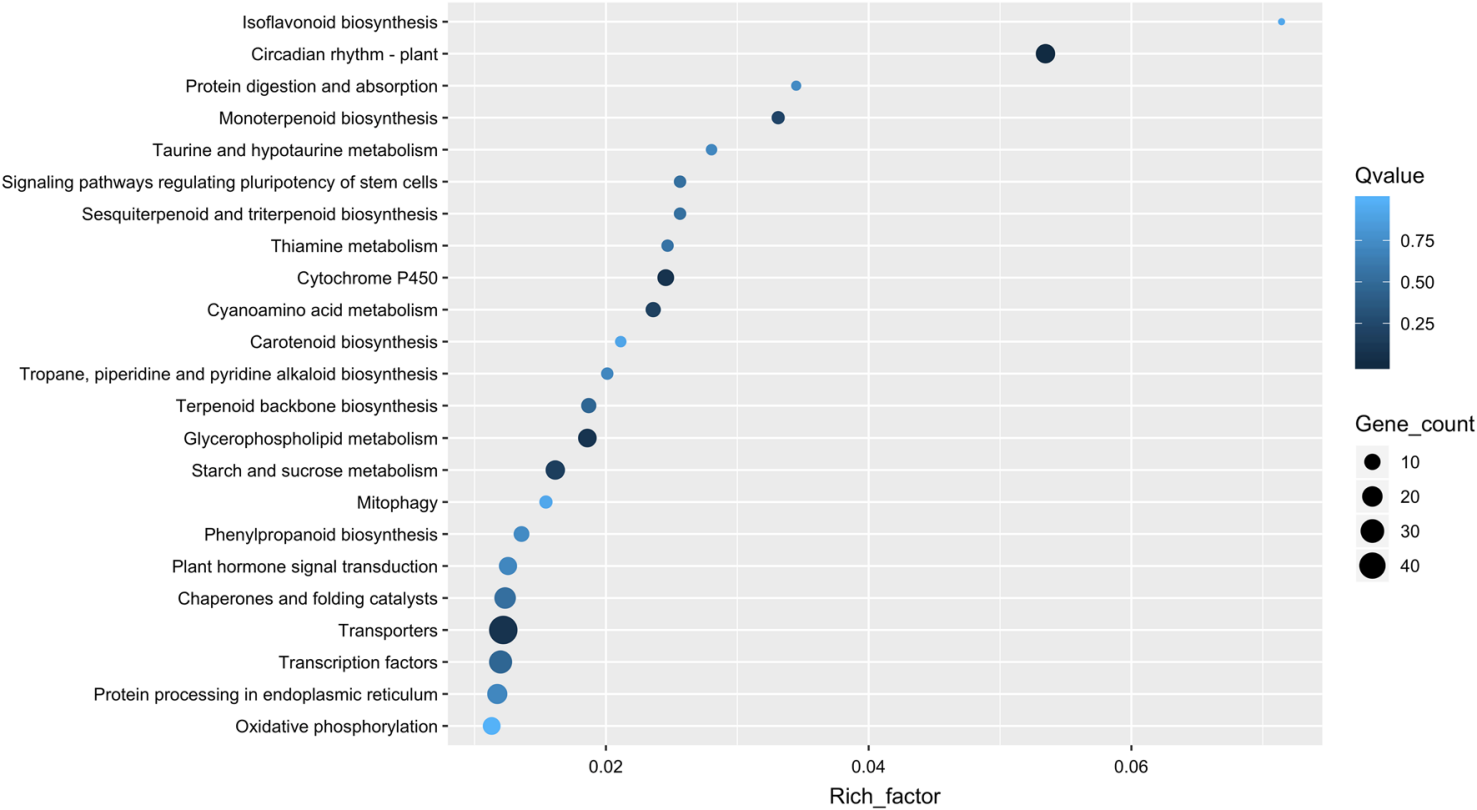
Scatter plot-enriched Kyoto Encyclopedia of Genes and Genomes (KEGG) pathways of differentially expressed genes (DEGs) in drought stressed plants compared to control plants in the field. The x-axis represents the gene ratio, which refers to the ratio of the DEG numbers annotated in the pathway term to all gene numbers annotated in the pathway term. The circle size indicates the number of DEGs that are associated with each significant pathway. The circle color indicates the significant level with the adjusted p-value using Benjamin Hochberg method.

KEGG pathway analysis was performed to identify the active biological pathways in the annotated guayule sequences. Upon mapping the annotated sequences, 40,917 transcripts (17.85%) were identified and assigned to 353 metabolic pathways, including ‘cellular processes’, ‘environmental information processing’, ‘genetic information processing’, ‘metabolism’ and ‘organismal systems’ (Fig. 3). Among these pathways, signal transduction (18,565 transcripts), carbohydrate metabolism (8768), and transport and catabolism (6294) were the three most represented. Fisher’s exact test on the identified 1677 differentially expressed genes (DEG) was performed to explore the changes in metabolic pathways under drought treatment (Fig. 4). Environmental adaptation, not surprisingly, was the category with the most differentially expressed transcripts. Interestingly, 1314 contigs mapped pathways involved in metabolism of terpenoids and polyketides, including monoterpenoid, sesquiterpenoid and triterpenoid, and terpenoid backbone biosynthesis. The expression of transcripts in this category were the second most differentially expressed by irrigation treatment. Terpenoids are highly abundant components of guayule resin. Isoflavonoid biosynthesis and circadian rhythm were among the topmost enriched pathways. Plant hormone signal transduction also showed a high enrichment score. Finally, the most enriched gene count among all pathways in our assembly encode a family of protein transporters. Aquaporins are water channels important for maintaining salt and water homeostasis, especially under biotic stress^45^. In agreement with Nelson et al.^46^, PIP1-3 (PaTc_178810) is one of the most induced contigs (FC = 12.18) in drought stressed guayule.

### Rubber and resin content in guayule are impacted differently by water irrigation levels

The guayule field irrigation study conducted in 2012–2015 in Maricopa, Arizona found consistently higher rubber content in plants treated with low (25% replacement of evapotranspiration (EVA), simulating drought conditions) irrigation water levels compared to the control (100% EVA) irrigation^6^ (Sup Fig. 1A). Stem subsamples from plants harvested in March 2015 were collected for the transcriptome analysis reported here. At that point drought-stressed plants had 8.6% rubber content, significantly higher than the 6.0% rubber in control plants (Sup Fig. 1B). Higher rubber content for guayule subjected to drought stress has been reported in greenhouse^47^ and field^6,36^ studies. The mechanism is unknown, but water stress could create less cell expansion due to the reduced turgor pressure, and smaller cell volume concentrating solutes in the cell, including substrate for rubber biosynthesis, providing a possible explanation to the observed higher rubber content in drought-stressed plants. Remarkably, Reddy and Das^48^ observed that low leaf water potential increased rubber transferase activity (and concomitant rubber content) in guayule.

In contrast, resin content was not correlated to irrigation level (Sup Fig. 1A) and was not significantly different between the two treatments (Sup Fig. 1B). Resin content in guayule is relatively insensitive to growing conditions such as seasonal temperature and water inputs^6,11,28,47,49^. It appears that, as a whole, unlike rubber, resin biosynthesis in guayule is less affected by the environment. However, guayule resin is a complex mixture^13,50^ and it is possible that biosynthesis of individual resin components such as terpenes may be impacted by water inputs or other environmental factors. Our transcriptome analysis found that many resin biosynthesis related transcripts were differentially expressed by drought (see section below), and therefore may provide breeding and genetic engineering targets for guayule improvement.

### The mevalonate pathway is mostly down-regulated in drought stressed guayule

**Figure 5:**
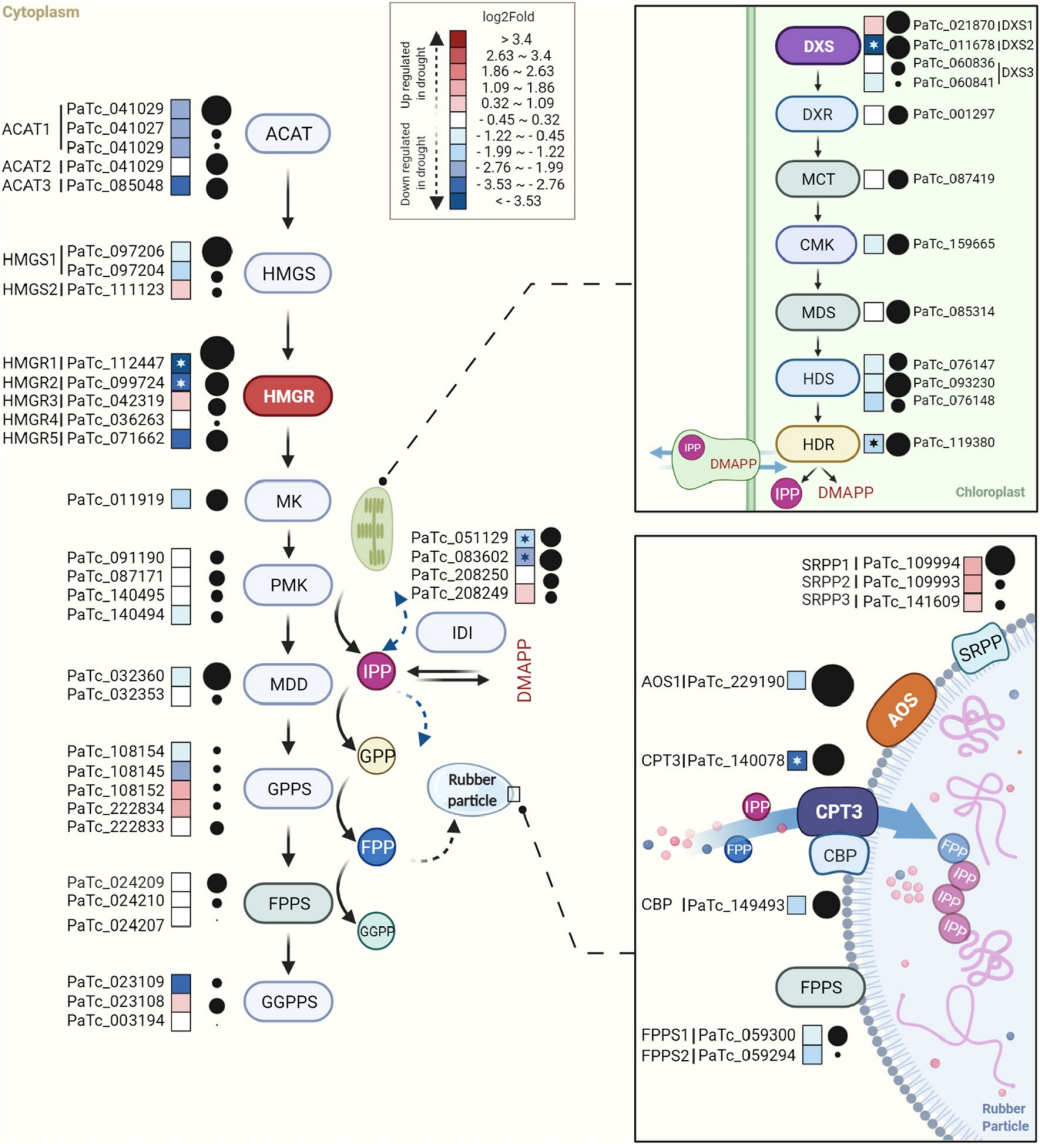
Expression of transcripts involved in mevalonate pathway (MVA), methylerythritol phosphate pathways (MEP) and rubber particle associated genes. Lower right, schematic model of the rubber biosynthetic machinery. Relative differential expression showed by a color gradient from low (blue) to high (red). Asterisks indicate significant differential expression (FDR < 0.05 and log2 fold-change > 2). Black dot sizes proportional to the expression level.

All isoprenoids, including NR, are derived from the precursor IPP. In plants, IPP is synthesized by two independent pathways: the mevalonate pathway (MVA) located mainly in the cytoplasm and the methylerythritol phosphate pathway (MEP) in plastids. Previous studies have shown that MVA pathway is likely the main source of IPP for rubber production in plants^51–53^. All genes encoding the MVA pathway enzymes were represented in our transcriptome (Table 2) however most of them (70%) were down-regulated by drought (Fig. 5). The enzyme 3-hydroxy-3-methylglutaryl-coenzyme A reductase (HMRG) is considered the key regulatory step in cytosolic IPP synthesis^54–56^. Our analysis identified five putative *HMGR* transcripts. Two *HMGR*-like transcripts were statistically significantly down-regulated (PaTc_112447 and PaTc_099724 with adjusted p-value at 0.0004 and 0.000, respectively). Interestingly, isoform PaTc_112447 (HMGR1 in Fig. 5), corresponds to a previously identified isoform from a guayule cold acclimated EST library^57^. No correlation between *HMGR* expression and rubber transferase activity was found in that study. As for the other three putative isoforms, one was moderately down-regulated (PaTc_071662), one slightly up-regulated (PaTc_042319) and another showed no change in expression level associated with plant water status (PaTc_036263). Activity of one of these five HMGR isoforms tracked with rubber transferase activity corresponding to an increase in rubber formation^58^. Identification of this rubber biosynthesis associated HMGR isoform will be critical not only for a better understanding of rubber biosynthetic pathway, but also to target this isoform, alongside other genes, in metabolic engineering and breeding efforts to increase rubber content in guayule.

**Table 2:** Identification and expression analysis of rubber biosynthesis associated genes.

Gene name	Contig	logFC	FDR	Ctrl_TMM	Drought_TMM	Accession no	Annotation
**Mevalonate (MVA) pathway**							
ACAT1	PaTc_041029	− 2.52	0.06	409.38	72.76	XP_024979490.1	Acetoacetyl-CoA thiolase
ACAT1	PaTc_041027	− 2.67	0.14	2.66	0.42	XP_024979490.1	Acetoacetyl-CoA thiolase
ACAT1	PaTc_041030	− 2.58	0.82	0.95	0.18	XP_024979490.1	Acetoacetyl-CoA thiolase
ACAT2	PaTc_085048	− 0.20	1.00	49.05	41.85	XP_022011037.1	Acetoacetyl-CoA thiolase
ACAT3	PaTc_107572	− 3.32	0.11	47.83	4.83	OTG07344.1	Acetoacetyl-CoA thiolase
HMGS1	PaTc_097206	− 1.17	0.74	336.44	145.22	XP_021969097.1	3-Hydroxy-3-methylglutaryl-coenzyme A synthase
HMGS1	PaTc_097204	− 1.51	0.73	4.87	1.69	XP_021969097.1	3-Hydroxy-3-methylglutaryl-coenzyme A synthase
HMGS2	PaTc_111123	0.55	0.95	1.74	2.56	XP_022022159.1	3-Hydroxy-3-methylglutaryl-coenzyme A synthase
HMGR1	PaTc_112447	− 4.34	0.00	849.24	42.90	XP_022016011.1	3-Hydroxy-3-methylglutaryl-coenzyme A reductase
HMGR2	PaTc_099724	− 2.92	0.00	104.71	13.74	ASJ80969.1	3-Hydroxy-3-methylglutaryl-coenzyme A reductase
HMGR3	PaTc_042319	0.91	0.87	8.50	16.58	XP_021981154.1	3-Hydroxy-3-methylglutaryl-coenzyme A reductase
HMGR4	PaTc_036263	0.25	1.00	0.86	1.04	XP_024990111.1	3-Hydroxy-3-methylglutaryl-coenzyme A reductase
HMGR5	PaTc_071662	− 3.39	0.06	55.53	5.59	XP_024974039.1	3-Hydroxy-3-methylglutaryl-coenzyme A reductase
MK	PaTc_011919	− 1.45	0.07	38.06	13.60	XP_021982774.1	mevalonate-5-kinase
PMK	PaTc_091190	0.00	1.00	6.32	6.37	XP_022029028.1	phosphomevalonate kinase
PMK	PaTc_087171	− 0.24	1.00	8.45	7.15	XP_021970137.1	phosphomevalonate kinase
PMK	PaTc_140495	0.11	1.00	3.43	3.70	XP_022029028.1	phosphomevalonate kinase
PMK	PaTc_140494	− 0.88	0.81	3.74	2.01	XP_022029028.1	phosphomevalonate kinase
MDD	PaTc_032360	− 0.67	0.95	249.84	155.84	XP_021993996.1	Diphosphomevalonate decarboxylase
MDD	PaTc_032353	− 0.30	1.00	3.12	2.54	XP_021993996.1	Diphosphomevalonate decarboxylase
IDI	PaTc_051129	− 1.78	0.02	29.22	8.46	XP_022033582.1	isopentenyl diphosphate isomerase
IDI	PaTc_083602	− 2.48	0.02	40.89	7.24	XP_021982603.1	isopentenyl diphosphate isomerase
IDI	PaTc_208250	0.06	1.00	8.02	7.96	XP_024990050.1	isopentenyl diphosphate isomerase
IDI	PaTc_208249	0.95	0.85	2.20	4.25	XP_024990050.1	isopentenyl diphosphate isomerase
**Rubber particle (RP) associated**							
AOS1	PaTc_229190	− 1.29	0.71	1138.35	445.44	XP_021983640.1	Allene oxide synthase
AOSL2	PaTc_122848	0.87	0.81	25.92	47.27	XP_021983640.1	Allene oxide synthase
AOSL3	PaTc_122845	− 0.42	1.00	34.35	25.58	XP_022034250.1	Allene oxide synthase
CBP	PaTc_149493	− 1.25	0.71	246.74	100.84	ATD87120.1	CPT-binding protein
CPT1	PaTc_044561	− 0.41	1.00	2.73	1.99	ATD87115.1	cis-prenyltransferases
CPT2	PaTc_069159	1.63	0.89	1.76	5.09	ATD87118.1	cis-prenyltransferases
CPT3	PaTc_140080	− 0.28	1.00	0.32	0.27	ATD87116.1	cis-prenyltransferases
CPT3	PaTc_140079	n.a	n.a	0.18	0.09	n.a	cis-prenyltransferases
CPT3	PaTc_140078	− 3.26	0.01	869.51	90.99	ATD87116.1	cis-prenyltransferases
SRPP1	PaTc_109994	1.16	0.19	192.50	421.14	AAQ11374.1	small rubber particle protein
SRPP2	PaTc_109993	1.51	0.94	0.70	2.15	AAQ11374.1	small rubber particle protein
SRPP3	PaTc_141609	0.92	0.53	159.87	298.83	AAQ11374.1	small rubber particle protein
FPPS1	PaTc_059300	− 0.80	0.94	20.41	11.89	O24241.1	farnesyl pyrophosphate synthase
FPPS2	PaTc_024209	− 0.07	1.00	26.16	24.70	O24242.1	farnesyl pyrophosphate synthase
FPPS	PaTc_059294	− 1.80	0.41	1.14	0.33	O24241.1	farnesyl pyrophosphate synthase
FPPS	PaTc_024210	0.00	1.00	2.20	2.18	O24242.1	farnesyl pyrophosphate synthase
FPPS	PaTc_024207	n.a	n.a	0.03	0.24	O24242.1	farnesyl pyrophosphate synthase
**Selected terpenoids pathway**							
GPPS	PaTc_108154	− 0.98	0.90	1.96	0.94	XP_022027531.1	geranyl pyrophosphate synthase
GPPS	PaTc_108145	− 2.39	0.55	1.44	0.26	XP_022027531.1	geranyl pyrophosphate synthase
GPPS	PaTc_108152	1.50	0.86	0.55	1.57	XP_022027531.1	geranyl pyrophosphate synthase
GPPS	PaTc_222834	1.38	0.90	0.68	1.74	XP_022027531.1	geranyl pyrophosphate synthase
GPPS	PaTc_222833	− 0.45	0.97	6.90	4.98	XP_022027531.1	geranyl pyrophosphate synthase
SQS	PaTc_219827	− 2.84	0.12	54.14	7.67	XP_022013547.1	squalene synthase
GGPPS	PaTc_023109	− 2.86	0.83	0.63	0.92	XP_022026732.1	geranylgeranyl pyrophosphate synthase
GGPPS	PaTc_023108	n.a	n.a	0.30	0.34	XP_022026732.1	geranylgeranyl pyrophosphate synthase
GGPPS	PaTc_003194	n.a	n.a	0.45	0.10	XP_022026732.1	geranylgeranyl pyrophosphate synthase

Interestingly, almost all MVA enzyme coding transcripts had at least one isoform unaffected by water treatment and/or another isoform slightly up-regulated. This diversity of transcriptional control suggests different MVA enzymes’ isoforms respond to different stimuli to exert specific metabolic control. The highest up-regulated transcript (PaTc_108152) encodes a geranyl pyrophosphate synthase (GPPS) isoform and the most down-regulated transcript encodes HMGR1 (Fig. 5). Overall our results indicate most of the MVA pathway in guayule is transcriptionally down-regulated by drought. It should be noted that isoprenoid pathway genes such as *HMGR*, 3-hydroxy-3-methylglutaryl-coenzyme A synthase (*HMGS*), phosphomevalonate kinase (*PMK*), isopentenyl diphosphate isomerase (*IDI*), and genaryl genaryl pyrophosphate synthase (*GGPPS*), have been reported to be down-regulated by drought in other plant species^59–61^.

Contigs coding all eight consecutive enzymes of the plastidic MEP pathway (Fig. 5) were identified (Supplementary Table S4). Of 129 contigs, 29 showed a higher expression trend under drought stress, however none was significant, while 55 showed a lower expression trend. Among the down-regulated contigs, 1-deoxy-D-xylulose 5-phosphate synthase (DXS), the rate limiting enzyme of MEP pathway was significantly down-regulated (Foldchange =  − 5.49). Down-regulation of 4‐hydroxy‐3‐methylbut‐2‐enyl diphosphate reductase (HDR) was also significant. MEP pathway analysis under drought stress in grape showed down-regulation of genes regulating the early pathway steps^62^.

It appears that at the transcription level, both MVA and MEP pathways are depressed by prolonged drought in guayule. In conifers, metabolic flux analysis found the MEP pathway was reduced by drought but much less than photosynthesis and transpiration, suggesting alternative carbon sources feed this metabolic pathway under drought stress^63^. A similar situation could apply to drought-stressed guayule; that is, an alternative source of carbon may be activated to provide the necessary synthesis of IPP and subsequently rubber.

### Is rubber biosynthesis under transcriptional control?

The RP is the site of NR production in plants and some fungi^64–66^; the polymer is synthesized by the membrane-associated RuT enzymatic complex. Two potentially essential members of the guayule RuT complex are a *cis*-prenyltransferase (CPT3) and a CPT-binding protein (CBP) which are hypothesized to form an active heteromer^29,67,68^. In addition to these proteins, the RuT complex may include two other RP-associated proteins: the small rubber particle protein (SRPP), and allene oxide synthase (AOS), both of indeterminate function but with a recognized indirect role in rubber biosynthesis^69–72^. Transcripts of all the above RP associated proteins were identified in our transcriptome (Table 2) and found to be mostly down-regulated by drought, with the exception of *SRPP*. Interestingly, the transcript encoding CPT3 (PaTc_140078), the specific isoform involved in rubber biosynthesis^67^ (*PaCPT3*) was found to be highly abundant in stem tissue as expected, but statistically significantly down-regulated by drought, in spite of the fact that drought-stressed guayule plants had higher rubber content (Fig. 5). The two other guayule *CPT* transcripts not involved in rubber biosynthesis^67^ (*PaCPT1* and *PaCPT2*) were of low abundance (Table 2), and were either slightly up-regulated (*PaCPT2*, PaTc_069159) or unaffected by plant water status (*PaCPT1*, PaTc_044561). These two CPTs are most likely involved in biosynthesis of dolichols^67,73^ and/or plastidial polyprenols essential for photosynthesis^74^. Transcripts encoding CBP (PaTc_149493) were also down-regulated by drought, although not to the extent of *CPT3* (Fig. 5). The *AOS* transcript PaTc_229190, encoding the most abundant protein associated with guayule RP^72,75,76^, not surprisingly had the highest expression levels among all other rubber biosynthesis related genes under both conditions (Table 2). Interestingly, this transcript was down-regulated 39% by drought despite the high rubber content. This negative correlation of AOS levels and rubber content was observed in *AOS*-silenced transgenic guayule lines, resulting in increased rubber content and higher RuT activity^72^. In that study, a structural role of AOS in guayule RP was proposed. Two other AOS isoforms (AOS-like, AOSL) of unknown function and localization have different expression profiles of slightly up-regulated (*AOSL2*, PaTc_122848) or no differential expression compared to control (*AOSL3*, PaTc_122845). The function of these AOSs could be the well-known role in jasmonic acid synthesis^77–79^.

The only RP-associated transcript found to be up-regulated was *SRPP* (PaTc_109994). SRPP is a stress response protein^80–82^ so it is not surprising drought would result in its up-regulation. Only one guayule *SRPP* gene has been cloned^69^ (guayule homolog of SRPP, GHS); our assembly identified two additional putative SRPP isoforms of unknown function but also up-regulated by drought (Table 2). Kajiura et al.^17^ antibodies used for RP proteins detection by western blot recognize an epitope present in GHS, and partially (92%) in the deduced protein sequences of the newly identified transcripts. The possibility of antibodies cross-reaction cannot be ruled out and therefore localization of the new SRPP isoforms remains to be determined. Although the role of SRPP in rubber biosynthesis remains to be elucidated, down-regulation of *SRPP* affected accumulation and quality of rubber in dandelion^70,71^. Dai et al.^32^ propose SRPP (and a related protein, rubber elongation factor (REF)) are likely negatively charged in the electrically neutral environment of Hevea latex, thus allowing the RP to maintain a stable colloidal form. Additionally, they propose SRPP and REF associates to the growing RP, allowing it to enlarge for the accumulation of new rubber molecules. SRPP may be needed to stabilize growing rubber particles in drought-stressed, high rubber-producing guayule.

Farnesyl pyrophosphate, required for initiation of rubber biosynthesis, is synthesized by farnesyl pyrophosphate synthase (FPPS) through condensation of genaryl pyrophosphate (GPP) and IPP. Two guayule *FPPS* genes have been cloned, characterized and their corresponding proteins confirmed to localize on the RP surface (Pan et al. 1996). Expression of *FPPS1* transcript (PaTc_059300) was slightly down-regulated by drought whereas that of *FPPS2* (PaTc_024209) appears to be unaffected (Fig. 5). Both transcripts are moderately abundant in stem tissue compared to three other putative isoforms identified in our assembly (Table 2).

**Figure 6:**
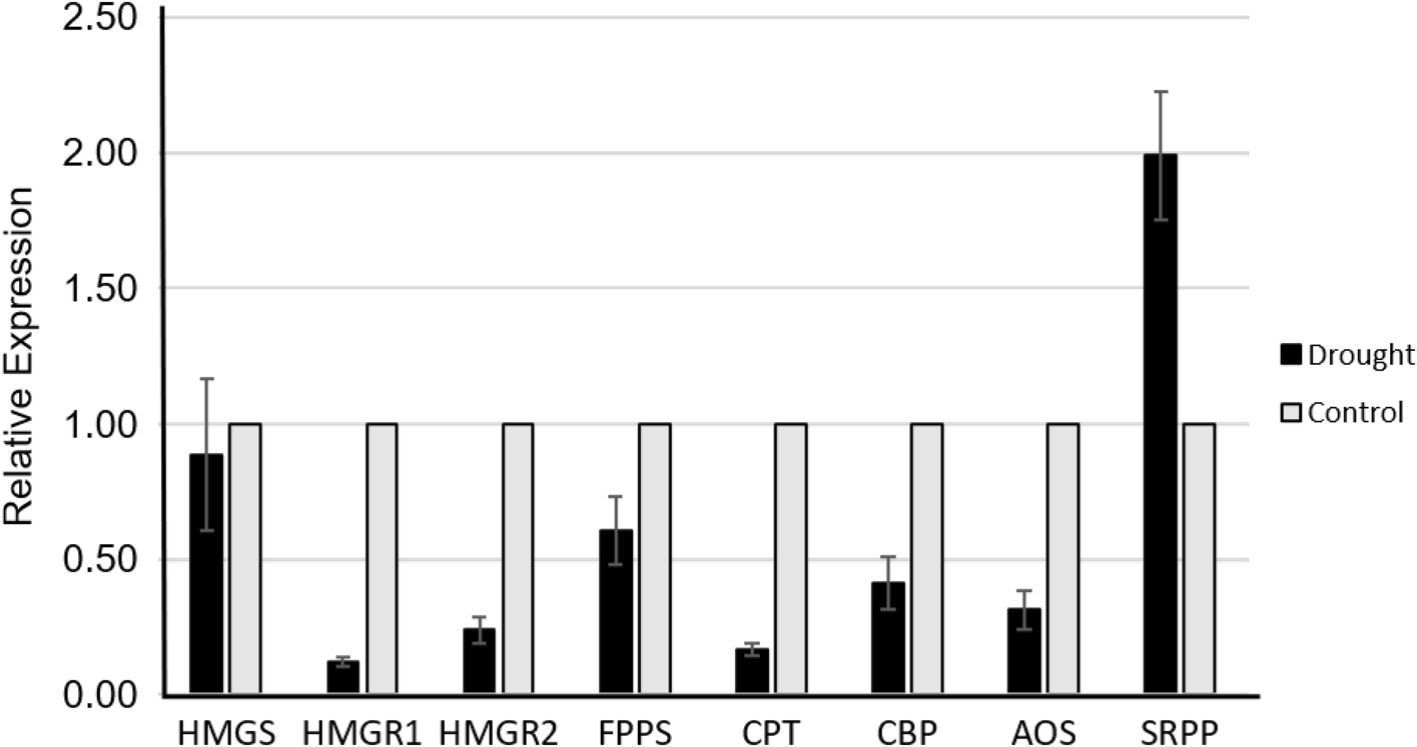
RT-PCR validation of expression levels. Selected guayule rubber biosynthesis related genes from stem tissues of plants grown under drought stress condition (black bars) relative to full irrigation control (grey bars). Values are the average of three biological replicates, error bars correspond to standard deviation error.

The fidelity of the in silico DEG predictions for rubber biosynthesis related genes was validated by quantitative reverse transcription PCR (qPCR). We analyzed the expression of *CPT3*, *CBP*, *AOS*, and *SRPP* as well as three MVA pathway genes (*HMGS*, *HMGR*, and *FPPS*, Fig. 6). In agreement with the DEG analysis, only SRPP was up-regulated under drought; all others were down-regulated compared to the control.

In summary, the low expression levels observed in rubber biosynthesis related transcripts (and the MVA pathway) contradict the high rubber content commonly found in drought-stressed guayule plants. These seemingly contradictory facts could be explained by the prospect that the rubber biosynthesis regulation is at the post-transcriptional, translational and/or post-translational levels. An earlier guayule field study^57^ failed to find a direct correlation between gene expression and RuT activity, also implying the control point of rubber biosynthesis in guayule may not be at the transcriptional level. Interestingly, drought-responsive long non-coding RNAs have been identified in guayule^43^, including one (GFTW01168370.1) featuring a conserved binding site for miR166, a drought-responsive microRNA.

Previously, under a laboratory environment, cold stress elicited expression of RP encoding genes in guayule including *AOS*, *CPT*, *FPPS* and *SRPP*^43^. Although both stresses (drought and cold) in guayule promote rubber synthesis, it appears that different genes in the biosynthetic pathway are expressed differently under each type of stress. Additionally, evidence suggests the coarse control of the MVA pathway is at the transcriptional level while the fine-tuning control at the post-transcriptional and/or post-translational levels^56^. In Hevea, proteomic analysis of latex found phosphorylation of some REF and SRPP isoforms^83^ following stimulation of rubber biosynthesis by ethylene treatment. Therefore, it is likely that both posttranscriptional and posttranslational mechanisms regulate rubber biosynthesis, as has been demonstrated for other plant secondary metabolites^84,85^.

### Drought has a mixed effect on expression levels of resin biosynthesis genes

**Figure 7:**
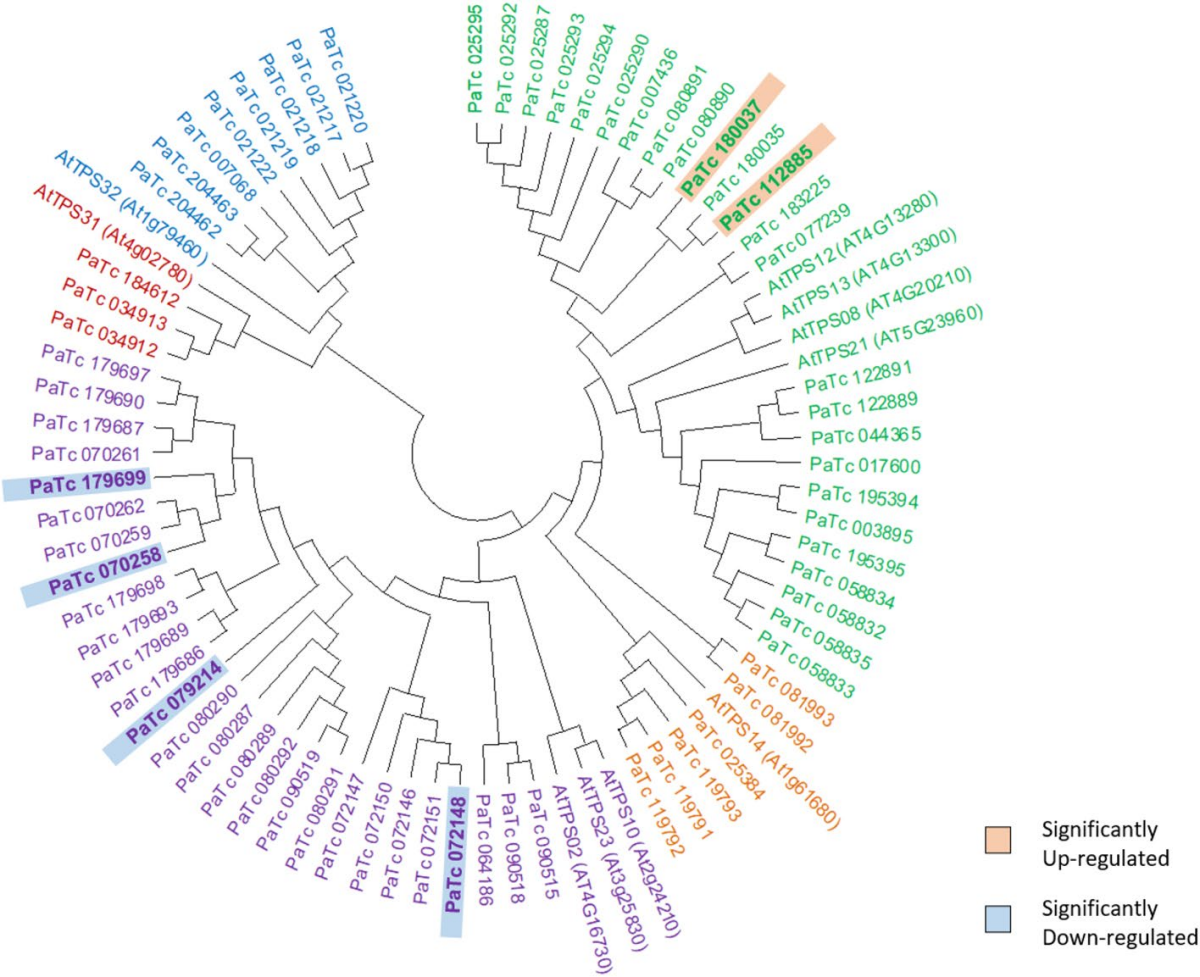
Molecular phylogenetic analysis of terpene synthases *TPSs* in stem tissue of field-grown guayule. The tree was constructed with MEGA X with maximum Likelihood method. Subfamilies highlighted by color: green (TPS-a), purple (TPS-b), red (TPS-c), blue (TPS-e/f) and orange (TPS-g) respectively. Significantly differentially expressed contigs in bold.

The largest class of compounds that make up guayule resin are terpenoids^12^ synthesized by terpene synthases (TPS). Using the highly conserved amino- and carboxyl- terminal domains, PF01397 and PF03936 respectively, we searched our assembly and identified a total of 70 contigs (Supplementary Table S5). Phylogenetic analysis (Fig. 7) clustered these contigs into 5 subfamilies with TPS-a and TPS-b the most expanded groups (26 and 27 contigs, respectively), comprising about 75% of the total TPSs. This is in accordance with other plant TPS profiles^86–88^. Previous studies have shown that *TPS* genes exhibit distinct tempo-spatial expression patterns with and without stress^86,88^. Among the 70 identified *TPS* in guayule, 15 of them showed no detectable transcript level in both control and drought-stressed plants for meaningful differential expression comparison. These *TPS* contigs are either nonfunctional pseudogenes or they are preferentially expressed in other organs. Among the other 55 contigs, which showed a differential expression pattern under drought stress, 21 contigs were up-regulated in the range of 0.23–5.53-fold, with an average fold change of 1.59; only two (PaTc_112885, PaTc_180037; Fig. 7) were significant, both involved in sesquiterpene synthesis. These two putative β-caryophyllene synthases likely synthesize a volatile terpene known to be induced when plants are subjected to different stresses, including wounding and herbivore attack^89^.

Thirty-four *TPS* contigs were suppressed under drought stress, in the range from 0.17 to 9.14-fold. Four of them (PaTc_070258, PaTc_079214, PaTc_179699, PaTc_072148; Fig. 7) were significantly down-regulated, all putative monoterpene synthases. These four contigs encode R-linalool synthase QH1 which synthesizes (3R)-linalool from GPP. Linalool is one of the most common monoterpenoids produced by plants and has been extensively investigated for its role in plant–insect interactions, including pollinator attraction^90^ and defense^91^. Notably, studies in *Solanum lycopersicum* and *Camellia sinensis* both showed significant decrease of linalool under drought^88,92^.

The most dramatically suppressed gene expression among the TPSs occurred to PaTc_079214 encoding a previously reported putative terpene synthase 3^46^. Interestingly, this transcript is one of the most significantly down regulated among the DEG set identified in this study.

The commercial viability of guayule as a crop depends on the successful utilization of resin as a high-value co-product. Our transcriptome offers a searchable database that can be mined to identify biosynthetic enzymes of the most abundant and high-value compounds including the guayulins and argentatins.

### Down-regulation of fructan synthesis by drought is accompanied by up-regulation of fructan depolymerization and other osmolytes biosynthesis genes

Fructan, a water-soluble polysaccharide, is the main reserve of carbohydrates in guayule^27^. Evidence shows fructans are actively involved in cold and drought stress response in plants by their capacity of maintaining cell membrane integrity through insertion in the lipid headgroup region of the membrane^25,26^. Two enzymes, sucrose:sucrose 1-fructosyl transferase (1-SST) and fructan:fructan 1-fructosyl transferase (1-FFT) are responsible for fructan synthesis, while the enzyme fructan 1-exohydrolase (1-FEH) catabolizes fructans^24^. In our assembly, contigs encoding *1Sst* (PaTc_199271, PaTc_199269) and *1Fft* (PaTc_016980) were both down-regulated significantly under drought stress, with an average fold change of 4.3 and 5.6 respectively, while *1-Feh* (PaTc_110775) was significantly induced (FC = 3.5). This suggests that drought-stressed guayule likely activated fructan depolymerization (Supplementary Table S6). In cereals, drought stress likewise led to fructan degradation^93,94^. Notably, these three genes showed the same expression pattern in cold-induced guayule stem tissue^43^ (Supplementary Table S6). Cold stress triggers rubber biosynthesis in guayule and the commonality of fructan metabolism transcriptional control in both drought and cold stress justifies a deeper analysis of fructans’ role in rubber biosynthesis as previously suggested by Benzioni and Mills^49^.

Interestingly, a similar gene expression pattern was observed in other carbohydrates metabolism (Supplementary Table S6). For example, fructose-bisphosphate aldolase (PaTc_033225), β-fructofuranosidase (PaTc_004249), GDP-L-galactose phosphorylase (PaTc_066454) and β-glucosidase (PaTc_068062), all of which are involved in hydrolyzing polysaccharides into simpler saccharides, were significantly induced under drought condition. On the other hand, glucose-1-phosphate adenylyltransferase (PaTc_166809) and raffinose synthase (PaTc_066497), both involved in polysaccharide synthesis, were down-regulated in stressed plants. Moreover, extensive studies have shown that polyamine (PA) is an important stress modulator in plants^95,96^. In our analyses, PA-synthesizing enzymes arginine decarboxylase (PaTc_082215) and S-adenosylmethionine decarboxylase (PaTc_065600, PaTc_003065, PaTc_118154) were heavily induced, while polyamine oxidase (PaTc_033142, PaTc_033141, PaTc_033146, PaTc_033145) were significantly down-regulated with average fold change of 3.5, suggesting a possible drought response strategy by maintaining a higher level of PA.

Taken together, it appears that osmotic stress in guayule triggers the accumulation of soluble sugars and PAs to help the plant cope with low water status. Additionally, drought generally suppresses photosynthesis and therefore there is less sugar available to support metabolic reactions such as rubber synthesis. Other sources of carbon such as carbohydrates’ catabolism could be providing the needed carbon for rubber synthesis.

## Conclusion

Our transcriptome analyses introduce a new perspective to expression of gene families related to key metabolic pathways in guayule stem tissue under drought stress. Results revealed that transcriptional status of genes involved in biosynthesis of the three major compounds, natural rubber, terpene resin, and fructan, are affected differently. Under drought conditions, the cytosolic isoprenoid biosynthetic pathway genes (MVA) and transcripts of RP associated proteins (including putative components of the RT complex) are mostly down-regulated in stem tissue, the main site of rubber synthesis in guayule, despite higher overall rubber content. This suggests the rubber biosynthesis control point is at the post-transcriptional level and beyond. Future engineering strategies for guayule, and possibly other rubber-producing crops, should carefully consider the growing body of transcriptomic information beyond metabolic pathway genes, as evidence of more complex regulation schemes emerge. Guayule resin biosynthesis transcripts, on the other hand, appear to be variably affected by water status and transcriptional control of individual resin compounds’ synthesis is likely unique. Finally, carbohydrate metabolism (in response to drought) is shown to be under transcriptional control in guayule, as is the case under cold stress, and under both conditions leading to enhanced rubber content in guayule.

## Methods

### Plant material and treatments

The plant material used was *Parthenium argentatum*, guayule, line AZ-3^97^, provided by the United States Department of Agriculture as publicly-curated germplasm (https://www.grin-global.org/),accession number PI 599676. Seeds were greenhouse planted, grown into seedlings, then transplanted into a field site at the University of Arizona, Maricopa Agricultural Center (MAC) and grown as described in Hunsaker et al.^6^. Following surface irrigation to establish the plants, subsurface drip irrigation was applied at 100% (control) and 25% (drought stress) of the soil water depletion, determined by field-calibrated, neutron moisture meters (Model 503, Campbell Pacific Nuclear, CPN, Martinez, CA). Plants were harvested in March 2015; rubber, resin, and biomass were quantified as per Hunsaker et al.^6^. Three biological replicates of bark tissue from stem were collected during the harvest. Tissues were immediately frozen in liquid nitrogen and stored at − 80 °C until RNA extraction. All germplasm sourcing, field operations, and laboratory procedures complied with relevant institutional, national, and international permissions, guidelines, and legislation.

### Library preparation and RNA-sequencing

Stem bark tissue (~ 2 g) from 29-month old guayule plants was the source of RNA for sequencing and qPCR analysis. Total RNA was extracted following Laudencia et al.^98^ protocol with the use of acid phenol:chloroform MB grade (Ambion, USA) instead of phenol:chloroform:isoamyl alcohol. The precipitated RNA was further cleaned with Qiagen RNeasy Plant Mini Kit (Qiagen, USA) and treated with DNA-*free*™ kit (Ambion, USA). PolyA-RNA was prepared employing Qiagen RNeasy/QIAshredder protocols (Qiagen, USA). RNA concentration was quantified with Quant-iT™ RiboGreen™ RNA Assay Kit (Thermofisher Scientific, USA). RNA quality was analyzed using 2100 Bioanalyzer (Agilent Technologies, USA). RNAseq library construction was carried out with KAPA Stranded RNA-Seq Library Preparation Kit Illumina® platforms (Kapa Biosystems, USA) per the manufacturer's instructions. RNAseq libraries with insert sizes of 200–500 bp, and sequenced using Illumina HiSeq 2000 platform with paired-end (PE) reads of 150 bp.

### De novo assembly and sequence processing

The raw reads were first cleaned by filtering out adaptor sequences and low-quality reads using Trimmomatic (v0.32). Trimmed reads, quality confirmed by running FastQC, were combined across six reads of both conditions. Both Trinity^99^ (v2.9.0) de novo and Trinity genome-guided (diploid guayule genome^37^) assemblies were supplied into the PASA pipeline^38^ to generate the comprehensive transcriptome database. To remove the redundant sequences, CD-HIT package was used for further clustering with a 300-bp sequence length and 95% similarity cut-off values. High-quality reads were mapped back to the assembled transcriptome sequences for validation. Reads were aligned using Bowtie-2 with default parameters.

### Gene function annotation

Gene functions were annotated against the Nr (NCBI non-redundant protein sequences), PFAM (protein family), Swiss-Prot (a manually annotated and reviewed protein sequence database), and TrEMBL (a computer-annotated protein sequence database) using local BLASTX program with an E-value threshold of 1e^−10^. GO analysis was performed using Blast2GO with the same E-value cutoff. Metabolic pathway mapping of the transcripts was performed using the KEGG Automatic Annotation Server (https://www.genome.jp/tools/kaas/). Plant transcription factors (TFs), transcriptional regulators (TRs) and protein kinases (PKs) were identified and classified into different gene families using standalone iTAK^100^ (v 18.12).

### Identification of differentially expressed genes (DEGs)

Differentially expressed transcripts under the two irrigation treatments were identified with edgeR^40^, using transcripts per million counts for each sample generated by Kallisto^39^. A two-fold change (FC ≥ 2) and FDR value < 0.05 were used to define the significant DEGs between treatment and control. GO and KEGG enrichment were performed on the transcripts identified as DEGs as well. For comparative analysis with cold stress guayule transcriptome, the raw data was acquired from NCBI BioProject PRJNA387289 with accession SRR5597223, SRR5597220, SRR5597221, SRR5597216, SRR5597215, SRR5597214, SRR5597224, SRR5597213, SRR5597212, SRR5597228, SRR5597231 AND SRR5597230 mapped back to TSA GFTW000000000.1^43^. Expression and differential expression analyses were completed in the same way as stated for the current drought study.

### Quantitative RT-PCR (qRT-PCR)

Total RNA from ground (~ 100 mg) stem bark tissue was extracted with TRIzol® reagent (Life Technologies, USA), cleaned with RNeasy Mini Kit (Qiagen, USA), and traces of DNA removed with DNA-free™ Kit (Life Technologies, USA). Two micrograms of total RNA were the template for oligo (dT)_20_-generated cDNA with SuperScriptIII First-Strand Synthesis System for qPCR (Life Technologies, USA) following manufacturer instructions. The qPCR reactions were carried out using Applied Biosystems 7500 Fast Real Time PCR System and SYBR Green chemistry (Life Technologies, USA) in 20 µl volume reactions containing 400 ng of template cDNA, 900 nM of each forward and reverse primer, 10 µl of Fast SYBR® Green Master Mix, and water as needed. The primers used are listed in Supplementary Table S7. Thermocycler temperature regime was: 95 °C for 20 s, followed by 40 cycles of 95 °C for 3 s and 60 °C for 30 s. Data were analyzed using the 7500 Fast System Detection Software (Life Technologies, USA) with manually set threshold. Expression of each target gene was calculated with the Livak and Schmittgen^101^ method, normalized to expression of the endogenous reference gene *eIF4a* or *18S*, and then to its expression in a calibrator (fully irrigated control plant). Three technical replicates reactions were run for each target gene, and the whole experiment was performed three times using the same RNA but freshly synthesized cDNA.

## Supplementary Information

Supplementary Information.

## Data Availability

The raw Illumina data generated in this study were deposited in the NCBI Sequence Read Archive (SRA) under the BioProject accession number PRJNA400611. The transcriptome assembly generated from the current study has been made available on the ARS Guayule genome website: Guayule Genomic Resources.
