# Author Correction: RNASeq analysis of drought-stressed guayule reveals the role of gene transcription for modulating rubber, resin, and carbohydrate synthesis

**DOI:** 10.1038/s41598-024-83264-z

**Published:** 2025-01-16

**Authors:** Chen Dong, Grisel Ponciano, Naxin Huo, Yong Gu, Daniel Ilut, Colleen McMahan

**Affiliations:** 1 USDA Agricultural Research Service, Western Regional Research Center, Albany, CA 94710 USA; 2 Plant Breeding and Genetics Section, School of Integrative Plant Science, Cornell University, Ithaca, NY 14853 USA

Correction to: *Scientific Reports* 10.1038/s41598-021-01026-7, published online 03 November 2021

The original version of this Article contained an error in the ‘Data availability’ section, where the Repository database was outdated.

“The raw Illumina data generated in this study were deposited in the NCBI Sequence Read Archive (SRA) under the BioProject accession number PRJNA400611. The transcriptome assembly generated from the current study has been made available on the A

RS Guayule genome website: https://probes.pw.usda.gov/Guayule/.”

now reads:

“The raw Illumina data generated in this study were deposited in the NCBI Sequence Read Archive (SRA) under the BioProject accession number PRJNA400611. The transcriptome assembly generated from the current study has been made available on the ARS Guayule genome website Guayule Genomic Resources.”

The original Article has been corrected.

