# Supplementary material for: RNASeq analysis of drought-stressed guayule reveals the role of gene transcription for modulating rubber, resin, and carbohydrate synthesis
Source: Sci Rep. 2021 Nov 3;11:21610. doi: 10.1038/s41598-021-01026-7 (PMC8566568; doi:10.1038/s41598-021-01026-7)
Supplement: Supplementary file 1 — Supplementary Information. [file 41598_2021_1026_MOESM1_ESM.pdf]

RNASeq analysis of drought-stressed guayule reveals the role of gene transcription for modulating rubber, resin, and carbohydrate synthesis

Chen Dong[1], Grisel Ponciano[1], Naxin Huo[1], Yong Gu[1], Daniel Ilut[2], Colleen McMahan[1]

USDA Agricultural Research Service, Western Regional Research Center, Albany, CA 94710, USA
Plant Breeding and Genetics Section, School of Integrative Plant Science, Cornell University, Ithaca, NY, 14853, USA

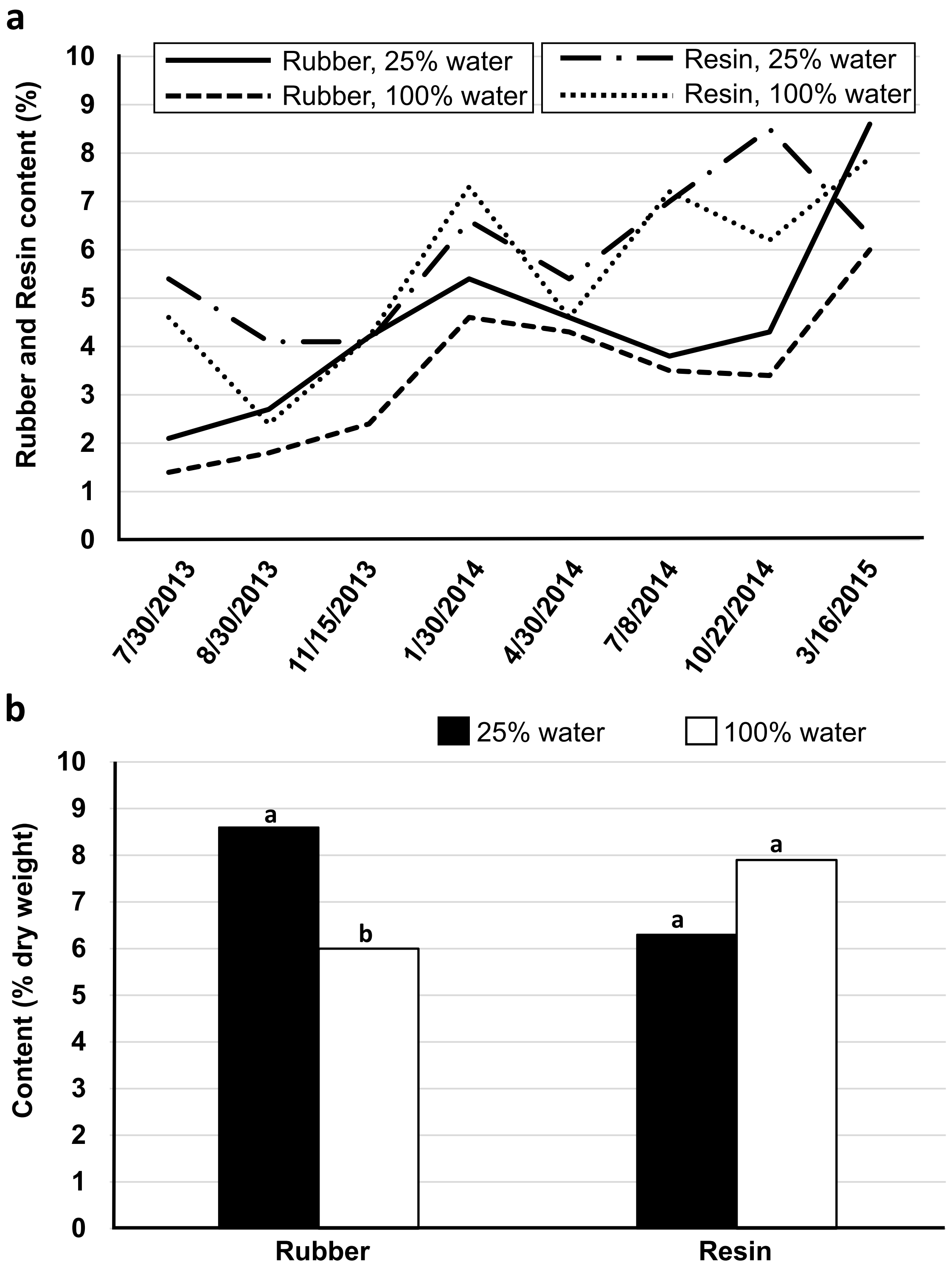

Sup. Figure 1. Rubber and Resin content of 29-month-old field-grown AZ-3 guayule plants. **(a)** throughout field trial, and **(b)** at harvest .

Table S1. Contigs annotated as Transcription Factors and differentially expressed

Contig ID	TF Family	Discription	logFC	FDR
PaTc_082629	AP2	related to AP2.7	-6.04	0.02
PaTc_086866	AP2	AP2 family protein	-5.13	0.00
PaTc_057689	ARF	auxin response factor 2	8.25	0.00
PaTc_005061	bHLH	LJRHL1-like 1	7.58	0.00
PaTc_032343	bHLH	phytochrome interacting factor 3	2.33	0.00
PaTc_116761	bHLH	bHLH family protein	-6.44	0.00
PaTc_172302	bHLH	phytochrome interacting factor 3	2.85	0.00
PaTc_016038	bZIP	abscisic acid responsive elements-binding factor 2	7.61	0.00
PaTc_071649	bZIP	basic leucine-zipper 44	-1.98	0.01
PaTc_112347	bZIP	basic leucine zipper 9	-8.74	0.00
PaTc_019718	C3H	C3H family protein	-3.22	0.00
PaTc_060589	C3H	C3H family protein	-7.31	0.00
PaTc_002287	CO-like	B-box type zinc finger protein with CCT domain	-11.27	0.00
PaTc_041802	CO-like	B-box type zinc finger protein with CCT domain	-2.32	0.04
PaTc_042661	CO-like	B-box type zinc finger protein with CCT domain	-4.81	0.01
PaTc_042662	CO-like	B-box type zinc finger protein with CCT domain	-10.81	0.00
PaTc_042663	CO-like	B-box type zinc finger protein with CCT domain	-2.89	0.00
PaTc_047157	CO-like	B-box type zinc finger protein with CCT domain	-2.54	0.02
PaTc_099176	CO-like	CONSTANS-like 5	2.21	0.02
PaTc_107801	CO-like	CONSTANS-like 5	3.38	0.00
PaTc_125806	CO-like	B-box type zinc finger protein with CCT domain	-3.14	0.02
PaTc_107065	Dof	Dof family protein	4.10	0.00
PaTc_024227	ERF	ethylene-responsive element binding factor 13	-2.25	0.04
PaTc_063563	ERF	ERF family protein	6.77	0.00
PaTc_038953	G2-like	G2-like family protein	-5.91	0.00
PaTc_038954	G2-like	G2-like family protein	-5.25	0.01
PaTc_221402	G2-like	G2-like family protein	-5.16	0.00
PaTc_221401	G2-like	G2-like family protein	-8.79	0.00
PaTc_221400	G2-like	G2-like family protein	-5.26	0.01
PaTc_078511	G2-like	phosphate starvation response 1	3.70	0.00
PaTc_012440	HD-ZIP	HD-ZIP family protein	2.94	0.01
PaTc_094068	HD-ZIP	HD-ZIP family protein	2.99	0.02
PaTc_032134	HRT-like	effector of transcription2	2.52	0.02
PaTc_001007	HSF	heat shock transcription factor A3	-6.64	0.00
PaTc_001009	HSF	heat shock transcription factor A3	-4.16	0.00
PaTc_075567	HSF	heat shock transcription factor B2A	-2.09	0.02
PaTc_107903	HSF	heat shock transcription factor C1	-3.05	0.01
PaTc_076451	MIKC_MADS	MIKC_MADS family protein	-2.08	0.05
PaTc_085983	MYB	myb domain protein 79	-4.35	0.02
PaTc_034764	MYB_related	MYB_related family protein	5.18	0.00
PaTc_054611	MYB_related	circadian clock associated 1	5.60	0.00
PaTc_054613	MYB_related	circadian clock associated 1	4.84	0.00
PaTc_054615	MYB_related	circadian clock associated 1	3.62	0.00
PaTc_060294	MYB_related	MYB_related family protein	3.40	0.00
PaTc_060297	MYB_related	MYB_related family protein	3.02	0.00
PaTc_097959	MYB_related	MYB_related family protein	5.07	0.00
PaTc_112574	MYB_related	MYB_related family protein	7.67	0.00
PaTc_112575	MYB_related	MYB_related family protein	7.21	0.00
PaTc_017784	NAC	NAC domain containing protein 52	-4.60	0.04
PaTc_196300	NAC	NAC domain containing protein 53	-2.15	0.02
PaTc_100386	NAC	NAC domain containing protein 57	-1.79	0.01
PaTc_115489	NAC	NAC domain containing protein 83	2.75	0.01
PaTc_127383	Nin-like	Nin-like family protein	2.08	0.04
PaTc_127385	Nin-like	Nin-like family protein	2.10	0.03
PaTc_036888	SBP	squamosa promoter binding protein-like 2	-2.30	0.01
PaTc_101732	SBP	squamosa promoter binding protein-like 10	8.11	0.00
PaTc_097463	TCP	TCP family protein	-5.49	0.02
PaTc_005126	WRKY	WRKY DNA-binding protein 28	2.36	0.01

Table S2. Comparision between cold and drought stress for contigs with siginificant differential expression

Cold ID	Cold_logFC	Cold_FDR	Pattern	Drought ID	Drought_FC	Drought_FDR	Pattern	Agree	Accession	Annotation
GFTW01069661.1	3.94	0.00	Up	PaTc_024875	3.94	0.00	Up	+	OTG15706.1	hypothetical protein HannXRQ_Chr09g0263341
GFTW01070838.1	2.61	0.00	Up	PaTc_205275	-2.46	0.00	Down	-	OTG29964.1	putative organ specific protein
GFTW01185174.1	-2.46	0.00	Down	PaTc_084926	-2.03	0.04	Down	+	XP_022037887.1	plasma membrane ATPase 4
GFTW01070783.1	2.59	0.00	Up	PaTc_205276	-2.81	0.00	Down	-	n.a	n.a
GFTW01082838.1	-1.93	0.00	Down	PaTc_197140	-2.95	0.00	Down	+	XP_021985787.1	cytochrome P450 81E8-like
GFTW01120663.1	-1.69	0.00	Down	PaTc_168046	-2.61	0.00	Down	+	XP_022012804.1	serine/threonine-protein kinase SRK2E-like
GFTW01133019.1	-2.39	0.00	Down	PaTc_113551	-2.37	0.04	Down	+	XP_021969425.1	probable purine permease 11 isoform X1
GFTW01086434.1	3.30	0.00	Up	PaTc_059116	4.88	0.02	Up	+	KVI00757.1	hypothetical protein Ccrd_020987
GFTW01162977.1	2.30	0.00	Up	PaTc_071706	-4.91	0.00	Down	-	XP_021975309.1	uncharacterized protein LOC110870436
GFTW01073738.1	-2.43	0.00	Down	PaTc_141536	-3.63	0.03	Down	+	XP_021998902.1	probable strigolactone esterase DAD2
GFTW01098050.1	-2.11	0.00	Down	PaTc_103326	2.47	0.01	Up	-	XP_022005929.1	cationic amino acid transporter 2, vacuolar-like isoform X2
GFTW01063600.1	-2.09	0.00	Down	PaTc_203473	-2.77	0.03	Down	+	XP_022009423.1	metal tolerance protein 4
GFTW01213880.1	-2.68	0.00	Down	PaTc_106026	2.11	0.04	Up	-	OTG16369.1	putative EF-hand domain pair
GFTW01081730.1	4.72	0.00	Up	PaTc_024877	3.33	0.01	Up	+	n.a	n.a
GFTW01159001.1	-3.07	0.00	Down	PaTc_093486	-4.23	0.02	Down	+	XP_022026446.1	lysine-specific demethylase JMJ30
GFTW01028039.1	2.72	0.00	Up	PaTc_041768	2.49	0.02	Up	+	PWA94387.1	CFIM-25-like protein
**GFTW01039094.1**	-4.27	0.00	Down	PaTc_135985	-4.78	0.00	Down	+	n.a	n.a
**GFTW01015007.1**	4.73	0.00	Up	PaTc_063563	6.77	0.00	Up	+	OTG18303.1	putative DNA-binding domain-containing protein
**GFTW01051834.1**	-3.49	0.00	Down	PaTc_125807	-4.74	0.02	Down	+	XP_021999985.1	zinc finger protein CONSTANS-LIKE 10-like
GFTW01094692.1	5.49	0.00	Up	PaTc_134822	2.72	0.04	Up	+	XP_022000588.1	thioredoxin-like 1-1, chloroplastic
**GFTW01039515.1**	-4.92	0.00	Down	PaTc_078511	3.70	0.00	Up	-	XP_021987337.1	protein PHOSPHATE STARVATION RESPONSE 1-like
GFTW01172165.1	-2.56	0.00	Down	PaTc_071392	-2.72	0.04	Down	+	XP_021987700.1	probable WRKY transcription factor 65
GFTW01126349.1	-1.70	0.00	Down	PaTc_028826	2.62	0.00	Up	-	XP_021982325.1	titin homolog isoform X8
GFTW01039791.1	-1.76	0.00	Down	PaTc_028826	-3.26	0.00	Down	+	XP_021982325.1	titin homolog isoform X8
GFTW01041594.1	-2.34	0.00	Down	PaTc_036487	4.09	0.00	Up	-	OTG22453.1	hypothetical protein HannXRQ_Chr06g0171661
GFTW01180950.1	1.60	0.00	Up	PaTc_054615	3.62	0.00	Up	+	XP_021983251.1	protein LHY-like
GFTW01171988.1	3.98	0.00	Up	PaTc_158534	2.94	0.02	Up	+	XP_022013412.1	serine carboxypeptidase-like 13 isoform X1
GFTW01103138.1	-5.65	0.00	Down	PaTc_074280	2.82	0.01	Up	-	XP_022038600.1	probable boron transporter 2
GFTW01167664.1	-2.29	0.00	Down	PaTc_018557	-5.41	0.02	Down	+	XP_022017894.1	two-component response regulator-like APRR1 isoform X2
GFTW01066771.1	2.04	0.00	Up	PaTc_199634	2.19	0.01	Up	+	XP_021976744.1	uncharacterized protein LOC110872280 isoform X1
GFTW01030225.1	3.22	0.00	Up	PaTc_033460	-2.99	0.00	Down	-	XP_022021908.1	sodium/hydrogen exchanger 4-like
GFTW01138612.1	-1.25	0.00	Down	PaTc_212688	-2.78	0.01	Down	+	XP_022034265.1	bifunctional nuclease 2-like
GFTW01081692.1	-2.07	0.00	Down	PaTc_018528	-3.78	0.00	Down	+	XP_022017894.1	two-component response regulator-like APRR1 isoform X2
GFTW01074528.1	1.80	0.00	Up	PaTc_099176	2.21	0.02	Up	+	XP_021996841.1	zinc finger protein CONSTANS-LIKE 5-like
GFTW01057676.1	-1.21	0.00	Down	PaTc_165972	-3.38	0.00	Down	+	n.a	n.a
GFTW01003619.1	2.52	0.00	Up	PaTc_002660	-4.06	0.00	Down	-	XP_023738354.1	EID1-like F-box protein 3
GFTW01150864.1	1.96	0.00	Up	PaTc_167104	2.81	0.01	Up	+	XP_022039242.1	uncharacterized protein LOC110941868
GFTW01024872.1	-1.35	0.00	Down	PaTc_019725	-2.95	0.01	Down	+	OTG30286.1	putative tetratricopeptide repeat (TPR)-like superfamily protein
GFTW01106055.1	-1.18	0.00	Down	PaTc_042663	-2.89	0.00	Down	+	OTG12171.1	putative B-box-type zinc finger, CCT domain protein
GFTW01066466.1	3.40	0.00	Up	PaTc_006730	3.16	0.02	Up	+	XP_022036842.1	tonoplast dicarboxylate transporter isoform X2
GFTW01165260.1	-1.65	0.00	Down	PaTc_038324	-2.01	0.01	Down	+	XP_024992719.1	calcium-transporting ATPase, endoplasmic reticulum-type
GFTW01115674.1	2.26	0.00	Up	PaTc_075567	-2.09	0.02	Down	-	XP_022006676.1	heat stress transcription factor B-2a-like
GFTW01131767.1	-1.65	0.00	Down	PaTc_028833	2.57	0.00	Up	-	XP_021982319.1	titin homolog isoform X2
GFTW01133258.1	3.99	0.00	Up	PaTc_107801	3.38	0.00	Up	+	XP_022037486.1	zinc finger protein CONSTANS-LIKE 3-like
GFTW01188898.1	-1.50	0.00	Down	PaTc_019724	-2.16	0.00	Down	+	OTG30286.1	putative tetratricopeptide repeat (TPR)-like superfamily protein
GFTW01156392.1	-1.96	0.00	Down	PaTc_022566	-3.29	0.00	Down	+	XP_022020727.1	zinc finger protein CONSTANS-LIKE 14-like
GFTW01058004.1	-4.11	0.00	Down	PaTc_059657	-4.27	0.00	Down	+	XP_022021139.1	indole-3-acetic acid-amido synthetase GH3.10-like
GFTW01180523.1	-1.50	0.00	Down	PaTc_057434	-3.34	0.00	Down	+	XP_022005564.1	agglutinin-like
GFTW01043266.1	-2.14	0.00	Down	PaTc_013666	2.50	0.00	Up	-	n.a	n.a
GFTW01069509.1	-1.40	0.00	Down	PaTc_022565	-3.61	0.00	Down	+	XP_022020727.1	zinc finger protein CONSTANS-LIKE 14-like
GFTW01166974.1	2.83	0.00	Up	PaTc_179959	-4.68	0.02	Down	-	XP_021980399.1	uncharacterized protein LOC110876537
GFTW01006873.1	-2.60	0.00	Down	PaTc_002288	-3.68	0.00	Down	+	XP_021998233.1	zinc finger protein CONSTANS-LIKE 9-like
GFTW01136110.1	-1.05	0.00	Down	PaTc_008411	-3.06	0.01	Down	+	XP_022039231.1	protochlorophyllide reductase, chloroplastic-like
GFTW01059656.1	-1.20	0.00	Down	PaTc_116643	-2.38	0.02	Down	+	XP_021973871.1	4-coumarate--CoA ligase-like 6
GFTW01117274.1	-2.87	0.00	Down	PaTc_030838	-5.76	0.00	Down	+	XP_021998149.1	BTB/POZ and TAZ domain-containing protein 4-like
GFTW01190116.1	2.07	0.00	Up	PaTc_147958	3.64	0.01	Up	+	XP_021990065.1	rop guanine nucleotide exchange factor 12-like
GFTW01129859.1	-1.44	0.00	Down	PaTc_038238	2.33	0.01	Up	-	XP_021982319.1	titin homolog isoform X2
GFTW01168404.1	3.22	0.00	Up	PaTc_192643	2.72	0.02	Up	+	XP_027094474.1	protein odr-4 homolog isoform X1

Table S3. Gene Set Enrichment Analysis (GSEA) of Gene Ontology (GO term)

Tags	GO ID	GO Name	GO Category	Size	ES	**NES**	Nominal p-	**FDR q-val**	FWER p-val	Rank at Ma	Leading Edge
[TOP]	GO:0015986	ATP synthesis coupled proton transpo	BIOLOGICAL_PROCESS	77	0.65	**2.29**	0	**0.000**	0	9602	tags=36%, list=11%, signal=41%
[BOTTOM]	GO:0010333	**terpene synthase activity**	MOLECULAR_FUNCTION	43	-0.69	**-2.25**	0	**0.000**	0	6750	tags=56%, list=8%, signal=60%
[TOP]	GO:0098869	**cellular oxidant detoxification**	BIOLOGICAL_PROCESS	101	0.61	**2.23**	0	**0.000**	0	15852	tags=42%, list=18%, signal=50%
[TOP]	GO:0004096	**catalase activity**	MOLECULAR_FUNCTION	34	0.75	**2.22**	0	**0.000**	0	15123	tags=65%, list=17%, signal=78%
[TOP]	GO:0006099	tricarboxylic acid cycle	BIOLOGICAL_PROCESS	81	0.61	**2.18**	0	**0.000**	0	20946	tags=56%, list=23%, signal=72%
[TOP]	GO:0006979	**response to oxidative stress**	BIOLOGICAL_PROCESS	111	0.60	**2.16**	0	**0.000**	0.001	6802	tags=26%, list=8%, signal=28%
[TOP]	GO:0022627	cytosolic small ribosomal subunit	CELLULAR_COMPONENT	69	0.63	**2.14**	0	**0.000**	0.002	19663	tags=48%, list=22%, signal=61%
[TOP]	GO:0046933	proton-transporting ATP synthase act	MOLECULAR_FUNCTION	41	0.70	**2.14**	0	**0.000**	0.003	10754	tags=46%, list=12%, signal=53%
[TOP]	GO:0006414	translational elongation	BIOLOGICAL_PROCESS	112	0.57	**2.10**	0	**0.000**	0.008	17503	tags=46%, list=19%, signal=58%
[TOP]	GO:0045261	proton-transporting ATP synthase cor	CELLULAR_COMPONENT	36	0.69	**2.06**	0	**0.000**	0.015	10754	tags=44%, list=12%, signal=50%
[TOP]	GO:0003746	translation elongation factor activity	MOLECULAR_FUNCTION	85	0.57	**2.02**	0	**0.001**	0.04	18641	tags=49%, list=21%, signal=62%
[TOP]	GO:0006096	glycolytic process	BIOLOGICAL_PROCESS	70	0.58	**2.01**	0	**0.001**	0.045	9726	tags=26%, list=11%, signal=29%
[TOP]	GO:0008553	proton-exporting ATPase activity, pho	MOLECULAR_FUNCTION	19	0.76	**2.00**	0	**0.001**	0.052	13026	tags=63%, list=14%, signal=74%
[TOP]	GO:0016717	**oxidoreductase activity, acting on pa**	MOLECULAR_FUNCTION	30	0.69	**1.98**	0.001946	**0.002**	0.072	21021	tags=67%, list=23%, signal=87%
[TOP]	GO:0033177	proton-transporting two-sector ATPas	CELLULAR_COMPONENT	60	0.59	**1.97**	0	**0.002**	0.09	15312	tags=40%, list=17%, signal=48%
[TOP]	GO:0042254	ribosome biogenesis	BIOLOGICAL_PROCESS	237	0.48	**1.96**	0	**0.002**	0.097	22392	tags=41%, list=25%, signal=55%
[TOP]	GO:0005743	mitochondrial inner membrane	CELLULAR_COMPONENT	194	0.50	**1.96**	0	**0.002**	0.105	15427	tags=34%, list=17%, signal=41%
[TOP]	GO:0007623	**circadian rhythm**	BIOLOGICAL_PROCESS	17	0.76	**1.95**	0	**0.002**	0.126	13263	tags=76%, list=15%, signal=90%
[TOP]	GO:0016832	aldehyde-lyase activity	MOLECULAR_FUNCTION	17	0.77	**1.94**	0	**0.002**	0.138	15668	tags=71%, list=17%, signal=85%
[TOP]	GO:0048038	quinone binding	MOLECULAR_FUNCTION	29	0.67	**1.94**	0	**0.003**	0.143	12748	tags=45%, list=14%, signal=52%
[TOP]	GO:0042744	**hydrogen peroxide catabolic process**	BIOLOGICAL_PROCESS	36	0.63	**1.93**	0.003623	**0.003**	0.177	15803	tags=42%, list=18%, signal=51%
[TOP]	GO:0006006	glucose metabolic process	BIOLOGICAL_PROCESS	57	0.58	**1.92**	0	**0.004**	0.209	19868	tags=42%, list=22%, signal=54%
[TOP]	GO:0020037	heme binding	MOLECULAR_FUNCTION	200	0.49	**1.91**	0	**0.004**	0.217	17235	tags=43%, list=19%, signal=52%
[TOP]	GO:0016620	**oxidoreductase activity, acting on th**	MOLECULAR_FUNCTION	67	0.57	**1.91**	0	**0.004**	0.245	12775	tags=37%, list=14%, signal=43%
[TOP]	GO:0006865	amino acid transport	BIOLOGICAL_PROCESS	33	0.65	**1.91**	0	**0.004**	0.247	3734	tags=30%, list=4%, signal=32%
[TOP]	GO:0005200	structural constituent of cytoskeleton	MOLECULAR_FUNCTION	44	0.61	**1.90**	0	**0.004**	0.266	7847	tags=30%, list=9%, signal=32%
[TOP]	GO:1990542	mitochondrial transmembrane transp	BIOLOGICAL_PROCESS	37	0.63	**1.89**	0	**0.005**	0.292	14879	tags=46%, list=17%, signal=55%
[TOP]	GO:0022625	cytosolic large ribosomal subunit	CELLULAR_COMPONENT	118	0.52	**1.89**	0	**0.005**	0.33	15901	tags=33%, list=18%, signal=40%
[TOP]	GO:0006119	oxidative phosphorylation	BIOLOGICAL_PROCESS	59	0.58	**1.88**	0	**0.005**	0.341	8772	tags=29%, list=10%, signal=32%
[TOP]	GO:0051536	iron-sulfur cluster binding	MOLECULAR_FUNCTION	146	0.50	**1.88**	0	**0.005**	0.342	15490	tags=34%, list=17%, signal=40%
[TOP]	GO:0006098	pentose-phosphate shunt	BIOLOGICAL_PROCESS	32	0.62	**1.86**	0.001923	**0.008**	0.467	10232	tags=34%, list=11%, signal=39%
[TOP]	GO:0019843	rRNA binding	MOLECULAR_FUNCTION	76	0.54	**1.84**	0	**0.009**	0.525	18818	tags=38%, list=21%, signal=48%
[TOP]	GO:0005618	cell wall	CELLULAR_COMPONENT	53	0.58	**1.84**	0	**0.010**	0.567	8091	tags=30%, list=9%, signal=33%

Table S4. Plastidic MEP pathway related genes and differential expression

Contig	logFC	FDR	Ctrl_TMM	Drought_TMM	Best-hit-A.th	Gene Name	Annotation
**PaTc_021870**	**0.43**	**1.00**	**33.95**	**44.06**	**AT2G26930**	**DXS**	**1-deoxy-D-xylulose5-phosphate synthase**
**PaTc_011678**	**-5.49**	**0.00**	**61.69**	**1.34**	**AT2G26930**	**DXS**	**1-deoxy-D-xylulose5-phosphate synthase**
PaTc_222887	-1.14	0.83	49.12	21.55	AT2G26930	DXS	1-deoxy-D-xylulose5-phosphate synthase
**PaTc_060836**	**-0.30**	**1.00**	**6.97**	**5.70**	**AT2G26930**	**DXS**	**1-deoxy-D-xylulose5-phosphate synthase**
**PaTc_060841**	**-0.58**	**1.00**	**0.76**	**0.53**	**AT2G26930**	**DXS**	**1-deoxy-D-xylulose5-phosphate synthase**
PaTc_222886	-1.84	0.63	11.00	3.03	AT2G26930	DXS	1-deoxy-D-xylulose5-phosphate synthase
PaTc_060846	-1.76	0.84	2.19	0.62	AT2G26930	DXS	1-deoxy-D-xylulose5-phosphate synthase
PaTc_060840	n.a.	n.a.	0.33	0.45	AT2G26930	DXS	1-deoxy-D-xylulose5-phosphate synthase
PaTc_068992	-1.85	0.64	12.81	3.42	AT2G26930	DXS	1-deoxy-D-xylulose5-phosphate synthase
PaTc_060833	n.a.	n.a.	0.00	0.00	AT2G26930	DXS	1-deoxy-D-xylulose5-phosphate synthase
PaTc_068993	0.00	1.00	0.43	0.43	AT2G26930	DXS	1-deoxy-D-xylulose5-phosphate synthase
PaTc_068995	n.a.	n.a.	1.48	0.00	AT2G26930	DXS	1-deoxy-D-xylulose5-phosphate synthase
PaTc_018130	-1.41	0.62	5.05	1.78	AT2G26930	DXS	1-deoxy-D-xylulose5-phosphate synthase
PaTc_060843	-1.10	0.97	1.00	0.47	AT2G26930	DXS	1-deoxy-D-xylulose5-phosphate synthase
PaTc_068996	-1.50	0.69	23.21	7.82	AT2G26930	DXS	1-deoxy-D-xylulose5-phosphate synthase
PaTc_068994	-0.29	1.00	1.10	0.89	AT2G26930	DXS	1-deoxy-D-xylulose5-phosphate synthase
PaTc_070209	n.a.	n.a.	0.99	0.00	AT2G26930	DXS	1-deoxy-D-xylulose5-phosphate synthase
PaTc_128149	n.a.	n.a.	0.63	0.45	AT2G26930	DXS	1-deoxy-D-xylulose5-phosphate synthase
PaTc_070208	n.a.	n.a.	0.58	0.19	AT2G26930	DXS	1-deoxy-D-xylulose5-phosphate synthase
PaTc_068997	-1.90	0.65	0.82	0.22	AT2G26930	DXS	1-deoxy-D-xylulose5-phosphate synthase
PaTc_134854	n.a.	n.a.	0.40	0.44	AT2G26930	DXS	1-deoxy-D-xylulose5-phosphate synthase
PaTc_025670	n.a.	n.a.	0.00	0.66	AT2G26930	DXS	1-deoxy-D-xylulose5-phosphate synthase
PaTc_018131	n.a.	n.a.	1.04	0.12	AT2G26930	DXS	1-deoxy-D-xylulose5-phosphate synthase
PaTc_025664	n.a.	n.a.	0.03	0.12	AT2G26930	DXS	1-deoxy-D-xylulose5-phosphate synthase
PaTc_025665	-0.06	1.00	1.54	1.49	AT2G26930	DXS	1-deoxy-D-xylulose5-phosphate synthase
PaTc_025667	n.a.	n.a.	0.36	0.32	AT2G26930	DXS	1-deoxy-D-xylulose5-phosphate synthase
PaTc_025666	n.a.	n.a.	0.19	0.13	AT2G26930	DXS	1-deoxy-D-xylulose5-phosphate synthase
PaTc_134855	n.a.	n.a.	0.30	0.26	AT2G26930	DXS	1-deoxy-D-xylulose5-phosphate synthase
PaTc_131895	1.70	0.83	1.22	3.78	AT2G26930	DXS	1-deoxy-D-xylulose5-phosphate synthase
PaTc_011681	n.a.	n.a.	1.60	0.11	AT2G26930	DXS	1-deoxy-D-xylulose5-phosphate synthase
PaTc_026531	0.08	1.00	25.01	26.09	AT2G26930	DXS	1-deoxy-D-xylulose5-phosphate synthase
PaTc_057943	-1.00	0.86	3.66	1.85	AT2G26930	DXS	1-deoxy-D-xylulose5-phosphate synthase
PaTc_057940	-0.78	0.64	49.20	27.98	AT2G26930	DXS	1-deoxy-D-xylulose5-phosphate synthase
PaTc_082456	-0.18	1.00	0.53	0.47	AT2G26930	DXS	1-deoxy-D-xylulose5-phosphate synthase
PaTc_089072	0.29	1.00	0.71	0.90	AT2G26930	DXS	1-deoxy-D-xylulose5-phosphate synthase
PaTc_089073	0.52	0.93	10.11	14.49	AT2G26930	DXS	1-deoxy-D-xylulose5-phosphate synthase
PaTc_124738	n.a.	n.a.	0.76	0.59	AT2G26930	DXS	1-deoxy-D-xylulose5-phosphate synthase
PaTc_082455	-0.11	1.00	20.99	19.25	AT2G26930	DXS	1-deoxy-D-xylulose5-phosphate synthase
PaTc_120250	-0.30	1.00	7.82	6.41	AT2G26930	DXS	1-deoxy-D-xylulose5-phosphate synthase
PaTc_015825	-0.51	1.00	3.92	2.72	AT2G26930	DXS	1-deoxy-D-xylulose5-phosphate synthase
PaTc_057942	-1.57	0.85	0.86	0.29	AT2G26930	DXS	1-deoxy-D-xylulose5-phosphate synthase
PaTc_057937	-1.06	0.96	0.49	0.24	AT2G26930	DXS	1-deoxy-D-xylulose5-phosphate synthase
PaTc_188506	1.25	0.93	3.83	8.87	AT2G26930	DXS	1-deoxy-D-xylulose5-phosphate synthase
PaTc_166320	1.87	0.56	0.75	2.69	AT2G26930	DXS	1-deoxy-D-xylulose5-phosphate synthase
PaTc_166321	0.76	0.99	28.38	46.80	AT2G26930	DXS	1-deoxy-D-xylulose5-phosphate synthase
PaTc_224061	n.a.	n.a.	0.46	0.40	AT2G26930	DXS	1-deoxy-D-xylulose5-phosphate synthase
**PaTc_001297**	**0.03**	**1.00**	**10.01**	**10.28**	**AT2G26930**	**DXR**	**1-deoxy-D-xylulose- 5-phosphate reductoisomerase**
PaTc_001296	-0.30	1.00	0.49	0.43	AT2G26930	DXR	1-deoxy-D-xylulose- 5-phosphate reductoisomerase
PaTc_001298	n.a.	n.a.	0.33	0.00	AT2G26930	DXR	1-deoxy-D-xylulose- 5-phosphate reductoisomerase
PaTc_143005	-1.35	0.29	41.49	16.24	AT2G26930	DXR	1-deoxy-D-xylulose- 5-phosphate reductoisomerase
PaTc_143001	-2.85	0.03	1.67	0.22	AT2G26930	DXR	1-deoxy-D-xylulose- 5-phosphate reductoisomerase
PaTc_143004	n.a.	n.a.	0.25	0.00	AT2G26930	DXR	1-deoxy-D-xylulose- 5-phosphate reductoisomerase
PaTc_143000	-1.90	0.28	2.80	0.73	AT2G26930	DXR	1-deoxy-D-xylulose- 5-phosphate reductoisomerase
PaTc_143003	n.a.	n.a.	0.79	0.07	AT2G26930	DXR	1-deoxy-D-xylulose- 5-phosphate reductoisomerase
PaTc_136212	n.a.	n.a.	0.65	0.26	AT2G26930	DXR	1-deoxy-D-xylulose- 5-phosphate reductoisomerase
PaTc_073642	-1.38	0.40	14.51	5.18	AT2G26930	DXR	1-deoxy-D-xylulose- 5-phosphate reductoisomerase
PaTc_176974	n.a.	n.a.	0.32	0.11	AT2G26930	DXR	1-deoxy-D-xylulose- 5-phosphate reductoisomerase
PaTc_001299	1.25	0.97	0.64	1.41	AT2G26930	DXR	1-deoxy-D-xylulose- 5-phosphate reductoisomerase
PaTc_143002	n.a.	n.a.	0.25	0.04	AT2G26930	DXR	1-deoxy-D-xylulose- 5-phosphate reductoisomerase
PaTc_096854	-1.37	0.66	8.31	3.00	AT2G26930	DXR	1-deoxy-D-xylulose- 5-phosphate reductoisomerase
PaTc_184629	n.a.	n.a.	0.36	1.72	AT2G26930	DXR	1-deoxy-D-xylulose- 5-phosphate reductoisomerase
PaTc_184628	1.42	0.92	0.44	1.13	AT2G26930	DXR	1-deoxy-D-xylulose- 5-phosphate reductoisomerase
**PaTc_087419**	**-0.34**	**0.99**	**19.27**	**14.93**	**AT4G34350**	**MCT**	**2-C-methyl-d-erythritol 4-phosphate cytidylyltransferase**
PaTc_087414	n.a.	n.a.	0.20	0.50	AT4G34350	MCT	2-C-methyl-d-erythritol 4-phosphate cytidylyltransferase
PaTc_087398	0.13	1.00	0.56	0.62	AT4G34350	MCT	2-C-methyl-d-erythritol 4-phosphate cytidylyltransferase
PaTc_087395	-1.94	0.22	0.69	0.19	AT4G34350	MCT	2-C-methyl-d-erythritol 4-phosphate cytidylyltransferase
PaTc_087381	n.a.	n.a.	0.44	0.13	AT4G34350	MCT	2-C-methyl-d-erythritol 4-phosphate cytidylyltransferase
PaTc_087406	n.a.	n.a.	0.37	0.05	AT4G34350	MCT	2-C-methyl-d-erythritol 4-phosphate cytidylyltransferase
**PaTc_159665**	**-0.48**	**0.93**	**23.80**	**16.82**	**AT2G26930**	**CMK**	**4-(cytidine 5'-diphospho)-2-C-methyl-d-erythritol kinase**
PaTc_159662	-2.22	0.85	1.31	0.28	AT2G26930	CMK	4-(cytidine 5'-diphospho)-2-C-methyl-d-erythritol kinase
PaTc_159663	n.a.	n.a.	0.42	0.52	AT2G26930	CMK	4-(cytidine 5'-diphospho)-2-C-methyl-d-erythritol kinase
PaTc_159664	0.69	0.93	0.95	1.46	AT2G26930	CMK	4-(cytidine 5'-diphospho)-2-C-methyl-d-erythritol kinase
PaTc_050407	-1.34	0.54	0.61	0.23	AT2G26930	CMK	4-(cytidine 5'-diphospho)-2-C-methyl-d-erythritol kinase
PaTc_073783	-1.49	0.41	2.40	0.87	AT2G26930	CMK	4-(cytidine 5'-diphospho)-2-C-methyl-d-erythritol kinase
PaTc_073787	-0.68	1.00	0.79	0.45	AT2G26930	CMK	4-(cytidine 5'-diphospho)-2-C-methyl-d-erythritol kinase
PaTc_073786	-0.41	1.00	0.94	0.67	AT2G26930	CMK	4-(cytidine 5'-diphospho)-2-C-methyl-d-erythritol kinase

PaTc_073790	-0.19	1.00	1.92	1.67	AT2G26930	CMK	4-(cytidine 5′-diphospho)-2-C-methyl-d-erythritol kinase
PaTc_073784	n.a.	n.a.	0.56	0.49	AT2G26930	CMK	4-(cytidine 5′-diphospho)-2-C-methyl-d-erythritol kinase
PaTc_073776	n.a.	n.a.	0.00	0.00	AT2G26930	CMK	4-(cytidine 5′-diphospho)-2-C-methyl-d-erythritol kinase
PaTc_073775	-1.14	0.75	1.46	0.65	AT2G26930	CMK	4-(cytidine 5′-diphospho)-2-C-methyl-d-erythritol kinase
PaTc_073781	n.a.	n.a.	0.00	0.00	AT2G26930	CMK	4-(cytidine 5′-diphospho)-2-C-methyl-d-erythritol kinase
PaTc_073779	n.a.	n.a.	0.18	0.03	AT2G26930	CMK	4-(cytidine 5′-diphospho)-2-C-methyl-d-erythritol kinase
PaTc_073774	-0.19	1.00	1.03	0.92	AT2G26930	CMK	4-(cytidine 5′-diphospho)-2-C-methyl-d-erythritol kinase
PaTc_073778	n.a.	n.a.	0.00	0.00	AT2G26930	CMK	4-(cytidine 5′-diphospho)-2-C-methyl-d-erythritol kinase
PaTc_073777	-1.71	0.83	1.46	0.42	AT2G26930	CMK	4-(cytidine 5′-diphospho)-2-C-methyl-d-erythritol kinase
PaTc_073782	n.a.	n.a.	0.23	0.31	AT2G26930	CMK	4-(cytidine 5′-diphospho)-2-C-methyl-d-erythritol kinase
PaTc_073789	n.a.	n.a.	0.09	0.24	AT2G26930	CMK	4-(cytidine 5′-diphospho)-2-C-methyl-d-erythritol kinase
**PaTc_085314**	**-0.36**	**0.98**	**52.43**	**39.47**	**AT4G34350**	**MDS**	**2-C-methyl-d-erythritol 2,4-cyclodiphosphate synthase**
PaTc_085316	-0.64	1.00	0.61	0.37	AT4G34350	MDS	2-C-methyl-d-erythritol 2,4-cyclodiphosphate synthase
PaTc_181823	n.a.	n.a.	0.34	0.46	AT4G34350	MDS	2-C-methyl-d-erythritol 2,4-cyclodiphosphate synthase
PaTc_085317	n.a.	n.a.	0.46	0.12	AT4G34350	MDS	2-C-methyl-d-erythritol 2,4-cyclodiphosphate synthase
**PaTc_076147**	**-1.11**	**0.19**	**17.35**	**8.15**	**AT5G60600**	**HDS**	**4-hydroxy-3-methylbut-2-enyl diphosphate synthase**
**PaTc_093230**	**-1.19**	**0.28**	**150.69**	**65.78**	**AT5G60600**	**HDS**	**4-hydroxy-3-methylbut-2-enyl diphosphate synthase**
**PaTc_076148**	**-1.85**	**0.12**	**8.57**	**2.35**	**AT5G60600**	**HDS**	**4-hydroxy-3-methylbut-2-enyl diphosphate synthase**
PaTc_152319	-0.91	0.60	30.67	15.96	AT5G60600	HDS	4-hydroxy-3-methylbut-2-enyl diphosphate synthase
PaTc_093232	-0.83	1.00	0.49	0.28	AT5G60600	HDS	4-hydroxy-3-methylbut-2-enyl diphosphate synthase
PaTc_059629	n.a.	n.a.	0.40	0.58	AT5G60600	HDS	4-hydroxy-3-methylbut-2-enyl diphosphate synthase
PaTc_165171	0.96	0.88	1.77	3.25	AT5G60600	HDS	4-hydroxy-3-methylbut-2-enyl diphosphate synthase
PaTc_059628	n.a.	n.a.	0.00	0.44	AT5G60600	HDS	4-hydroxy-3-methylbut-2-enyl diphosphate synthase
PaTc_123869	-0.99	0.63	71.18	27.73	AT5G60600	HDS	4-hydroxy-3-methylbut-2-enyl diphosphate synthase
PaTc_093231	-1.27	0.74	1.70	0.68	AT5G60600	HDS	4-hydroxy-3-methylbut-2-enyl diphosphate synthase
PaTc_206952	n.a.	n.a.	0.96	1.13	AT5G60600	HDS	4-hydroxy-3-methylbut-2-enyl diphosphate synthase
**PaTc_119380**	**-1.43**	**0.04**	**108.97**	**40.16**	**AT2G26930**	**HDR**	**4-hydroxy-3-methylbut-2-enyl diphosphate reductase**
PaTc_017844	-2.25	0.30	38.32	7.85	AT2G26930	HDR	4-hydroxy-3-methylbut-2-enyl diphosphate reductase
PaTc_059924	2.38	0.24	18.73	92.94	AT2G26930	HDR	4-hydroxy-3-methylbut-2-enyl diphosphate reductase
PaTc_051256	-0.24	1.00	2.22	1.85	AT2G26930	HDR	4-hydroxy-3-methylbut-2-enyl diphosphate reductase
PaTc_017843	-3.94	0.16	5.65	0.35	AT2G26930	HDR	4-hydroxy-3-methylbut-2-enyl diphosphate reductase
PaTc_119378	-1.85	0.11	2.24	0.62	AT2G26930	HDR	4-hydroxy-3-methylbut-2-enyl diphosphate reductase
PaTc_177750	2.08	0.50	22.68	88.92	AT2G26930	HDR	4-hydroxy-3-methylbut-2-enyl diphosphate reductase
PaTc_059921	3.04	0.57	0.20	1.59	AT2G26930	HDR	4-hydroxy-3-methylbut-2-enyl diphosphate reductase
PaTc_119379	0.15	1.00	0.36	0.40	AT2G26930	HDR	4-hydroxy-3-methylbut-2-enyl diphosphate reductase
PaTc_119382	n.a.	n.a.	0.00	0.00	AT2G26930	HDR	4-hydroxy-3-methylbut-2-enyl diphosphate reductase
PaTc_119377	-1.19	0.69	2.53	1.10	AT2G26930	HDR	4-hydroxy-3-methylbut-2-enyl diphosphate reductase
PaTc_059925	1.30	0.98	8.90	19.89	AT2G26930	HDR	4-hydroxy-3-methylbut-2-enyl diphosphate reductase
PaTc_017845	n.a.	n.a.	0.13	0.00	AT2G26930	HDR	4-hydroxy-3-methylbut-2-enyl diphosphate reductase
PaTc_119381	-1.31	0.11	161.12	58.68	AT2G26930	HDR	4-hydroxy-3-methylbut-2-enyl diphosphate reductase
PaTc_051257	n.a.	n.a.	0.10	0.17	AT2G26930	HDR	4-hydroxy-3-methylbut-2-enyl diphosphate reductase
PaTc_037082	2.16	0.58	1.76	7.04	AT2G26930	HDR	4-hydroxy-3-methylbut-2-enyl diphosphate reductase
PaTc_177746	0.07	1.00	73.54	68.89	AT2G26930	HDR	4-hydroxy-3-methylbut-2-enyl diphosphate reductase
PaTc_107108	n.a.	n.a.	0.25	0.13	AT2G26930	HDR	4-hydroxy-3-methylbut-2-enyl diphosphate reductase
PaTc_167006	n.a.	n.a.	0.60	0.32	AT2G26930	HDR	4-hydroxy-3-methylbut-2-enyl diphosphate reductase
PaTc_107107	n.a.	n.a.	0.25	0.06	AT2G26930	HDR	4-hydroxy-3-methylbut-2-enyl diphosphate reductase
PaTc_107106	n.a.	n.a.	0.08	0.17	AT2G26930	HDR	4-hydroxy-3-methylbut-2-enyl diphosphate reductase
PaTc_064595	n.a.	n.a.	0.34	0.25	AT2G26930	HDR	4-hydroxy-3-methylbut-2-enyl diphosphate reductase
PaTc_167003	n.a.	n.a.	0.09	0.00	AT2G26930	HDR	4-hydroxy-3-methylbut-2-enyl diphosphate reductase
PaTc_167005	n.a.	n.a.	0.16	0.11	AT2G26930	HDR	4-hydroxy-3-methylbut-2-enyl diphosphate reductase
PaTc_167007	n.a.	n.a.	0.48	0.15	AT2G26930	HDR	4-hydroxy-3-methylbut-2-enyl diphosphate reductase
PaTc_167004	n.a.	n.a.	0.12	0.10	AT2G26930	HDR	4-hydroxy-3-methylbut-2-enyl diphosphate reductase
PaTc_167008	n.a.	n.a.	0.16	0.09	AT2G26930	HDR	4-hydroxy-3-methylbut-2-enyl diphosphate reductase

Table S5. Identification of guayule TPS gene family (Statistically significant expression highlighted bold)

Contig ID	Domain	# aa	Subfamily	logFC	FDR	Gene ID	Annotation	Ctrl_TMM	Drought_TMM
PaTc_003895	PF01397	311	TPS-a			XP_022028638.1	alpha-copaene synthase-like	0.07	0.06
PaTc_007068	PF01397	214	TPS-e/f			XP_022027300.1	ent-kaur-16-ene synthase, chloroplastic-like isoform X1	0.35	0.34
PaTc_007436	PF01397	155	TPS-a			AMP42987.1	germacrene D	0.00	0.34
PaTc_021217	PF01397 and PF03936	435	TPS-e/f	2.39	0.79	XP_022018100.1	ent-kaur-16-ene synthase, chloroplastic-like	0.10	0.52
PaTc_021218	PF01397 and PF03936	791	TPS-e/f	2.00	0.11	XP_022018100.1	ent-kaur-16-ene synthase, chloroplastic-like	0.95	3.87
PaTc_021220	PF01397 and PF03936	423	TPS-e/f	-0.42	1.00	XP_022018100.1	ent-kaur-16-ene synthase, chloroplastic-like	1.57	1.12
PaTc_021222	PF01397 and PF03936	722	TPS-e/f	-7.83	0.18	XP_022018100.1	ent-kaur-16-ene synthase, chloroplastic-like	0.67	0.00
PaTc_025287	PF01397 and PF03936	549	TPS-a	-0.95	0.93	XP_021969440.1	(-)-germacrene D synthase-like	93.73	49.23
PaTc_025292	PF01397 and PF03936	294	TPS-a	-1.87	0.79	AMP42987.1	germacrene D	6.59	1.89
PaTc_025294	PF01397	327	TPS-a	-1.95	0.35	AMP42987.1	germacrene D	5.18	1.36
PaTc_025295	PF01397	462	TPS-a	-1.94	0.18	XP_021969440.1	(-)-germacrene D synthase-like	17.25	4.54
PaTc_034912	PF01397	314	TPS-c			OTG30196.1	putative terpenoid cyclases/protein prenyltransferase alpha-alpha toroid	0.20	0.16
PaTc_034913	PF01397 and PF03936	804	TPS-c	0.44	1.00	OTG30196.1	putative terpenoid cyclases/protein prenyltransferase alpha-alpha toroid	0.37	0.49
PaTc_044365	PF01397	102	TPS-a			AJT60315.1	germacrene A synthase	0.00	0.00
PaTc_058832	PF01397 and PF03936	579	TPS-a	0.31	1.00	XP_022017847.1	alpha-copaene synthase-like	0.67	0.86
PaTc_058833	PF01397	400	TPS-a	0.86	1.00	XP_022017847.1	alpha-copaene synthase-like	0.95	1.78
PaTc_058834	PF01397 and PF03936	306	TPS-a	1.79	0.74	XP_022028638.1	alpha-copaene synthase-like	1.14	4.00
PaTc_058835	PF01397	497	TPS-a	1.34	0.71	XP_022017847.1	alpha-copaene synthase-like	0.35	0.92
PaTc_064186	PF01397	444	TPS-b	3.41	0.13	XP_021973238.1	R-linalool synthase QH1, chloroplastic-like	0.13	1.45
**PaTc_070258**	**PF01397 and PF03936**	**593**	**TPS-b**	**-3.16**	**0.02**	**XP_022000734.1**	**R-linalool synthase QH1, chloroplastic-like**	**6.99**	**0.76**
PaTc_070259	PF01397 and PF03936	500	TPS-b	-2.70	0.86	XP_022000734.1	R-linalool synthase QH1, chloroplastic-like	1.45	0.23
PaTc_072146	PF01397	312	TPS-b	-4.08	0.14	AMP42989.1	putative monoterpene synthase	13.04	0.78
PaTc_072147	PF01397	423	TPS-b	-3.55	0.30	AMP42989.1	putative monoterpene synthase	12.76	1.08
PaTc_072150	PF01397	553	TPS-b	-4.51	0.29	AMP42989.1	putative monoterpene synthase	7.93	0.34
PaTc_072151	PF01397 and PF03936	591	TPS-b	-3.52	0.85	AMP42989.1	putative monoterpene synthase	10.49	0.91
PaTc_077239	PF01397 and PF03936	561	TPS-a			OTF85122.1	putative terpenoid cyclases/protein prenyltransferase alpha-alpha toroid	0.37	0.13
**PaTc_079214**	**PF01397 and PF03936**	**588**	**TPS-b**	**-9.14**	**0.00**	**XP_022028257.1**	**R-linalool synthase QH1, chloroplastic-like**	**36.14**	**0.05**
PaTc_080287	PF01397	231	TPS-b			XP_022000722.1	R-linalool synthase QH1, chloroplastic-like	0.00	0.00
PaTc_080289	PF01397	109	TPS-b			XP_022000722.1	R-linalool synthase QH1, chloroplastic-like	0.46	0.00
PaTc_080290	PF01397	182	TPS-b			XP_022000722.1	R-linalool synthase QH1, chloroplastic-like	0.19	0.09
PaTc_080291	PF01397	146	TPS-b			XP_022000722.1	R-linalool synthase QH1, chloroplastic-like	0.14	0.00
PaTc_080292	PF01397	182	TPS-b			XP_022000722.1	R-linalool synthase QH1, chloroplastic-like	0.14	0.06
PaTc_080890	PF01397	546	TPS-a	1.91	0.36	XP_021969440.1	(-)-germacrene D synthase-like	15.88	59.50
PaTc_080891	PF01397	323	TPS-a	0.85	1.00	XP_021969440.1	(-)-germacrene D synthase-like	3.81	6.82
PaTc_081992	PF01397	537	TPS-g	-0.27	1.00	XP_022008887.1	probable terpene synthase 11 isoform X2	1.19	0.99
PaTc_081993	PF01397	370	TPS-g	-0.78	1.00	XP_022008887.1	probable terpene synthase 11 isoform X2	0.55	0.33
PaTc_090515	PF01397	218	TPS-b	-2.15	0.81	PWA35421.1	myrcene synthase protein	60.69	14.56
PaTc_090518	PF01397	237	TPS-b	-2.07	0.85	XP_023767623.1	R-linalool synthase QH1, chloroplastic-like	71.59	18.23
PaTc_090519	PF01397	177	TPS-b	-3.37	0.62	PWA35421.1	myrcene synthase protein	10.42	1.02
**PaTc_112885**	**PF01397**	**548**	**TPS-a**	**3.50**	**0.00**	**PWA41248.1**	**Beta-caryophyllene synthase**	**0.87**	**9.84**
PaTc_119791	PF01397	521	TPS-g	-2.26	0.15	XP_022016226.1	(3S,6E)-nerolidol synthase 1-like	1.86	0.37
PaTc_119792	PF01397	434	TPS-g	-3.10	0.67	XP_022016226.1	(3S,6E)-nerolidol synthase 1-like	1.13	0.12
PaTc_119793	PF01397	399	TPS-g			XP_022017131.1	(3S,6E)-nerolidol synthase 1-like	0.22	0.17
PaTc_122889	PF01397	559	TPS-a	-1.63	0.98	AJT60315.1	germacrene A synthase	9.42	3.17
PaTc_122891	PF01397	465	TPS-a	1.16	0.96	AJT60315.1	germacrene A synthase	0.50	1.16
PaTc_134393	PF01397	602	TPS-	-1.85	0.73	OTG15111.1	putative terpenoid cyclases/protein prenyltransferase alpha-alpha toroid	10.71	3.12
PaTc_179686	PF01397	596	TPS-b	2.00	0.56	XP_022009132.1	R-linalool synthase QH1, chloroplastic-like	12.22	47.20
PaTc_179687	PF01397	347	TPS-b			XP_022000736.1	R-linalool synthase QH1, chloroplastic-like	0.32	0.02
PaTc_179689	PF01397	520	TPS-b	1.13	0.93	XP_022009132.1	R-linalool synthase QH1, chloroplastic-like	8.16	16.74
PaTc_179690	PF01397	548	TPS-b	0.95	1.00	XP_022000734.1	R-linalool synthase QH1, chloroplastic-like	0.26	0.50
PaTc_179693	PF01397	551	TPS-b	1.47	0.94	XP_022009132.1	R-linalool synthase QH1, chloroplastic-like	0.80	2.19
PaTc_179697	PF01397	455	TPS-b	-1.81	0.73	XP_022000734.1	R-linalool synthase QH1, chloroplastic-like	1.92	0.52
PaTc_179698	PF01397	316	TPS-b	0.70	1.00	XP_022009132.1	R-linalool synthase QH1, chloroplastic-like	1.83	2.81
**PaTc_179699**	**PF01397**	**500**	**TPS-b**	**-2.90**	**0.02**	**XP_022000736.1**	**R-linalool synthase QH1, chloroplastic-like**	**23.06**	**3.06**
PaTc_180035	PF01397	547	TPS-a	-1.16	0.99	XP_021985836.1	beta-caryophyllene synthase-like	20.16	8.91
**PaTc_180037**	**PF01397**	**546**	**TPS-a**	**5.53**	**0.00**	**XP_021995087.1**	**beta-caryophyllene synthase-like**	**0.39**	**18.47**
PaTc_183225	PF01397	575	TPS-a	-0.62	1.00	OTF92560.1	putative terpenoid cyclases/protein prenyltransferase alpha-alpha toroid	7.85	4.78
PaTc_184612	PF01397	798	TPS-c	-1.97	0.82	XP_022010671.1	ent-copalyl diphosphate synthase, chloroplastic-like	1.54	0.38
PaTc_195394	PF01397	578	TPS-a	0.97	0.79	XP_022028638.1	alpha-copaene synthase-like	1.75	3.47
PaTc_195395	PF01397	307	TPS-a			XP_022028638.1	alpha-copaene synthase-like	0.90	0.00
PaTc_204462	PF01397	796	TPS-e/f	0.23	1.00	XP_022027301.1	ent-kaur-16-ene synthase, chloroplastic-like isoform X2	0.87	1.02
PaTc_204463	PF01397	529	TPS-e/f	-0.17	1.00	XP_022027301.1	ent-kaur-16-ene synthase, chloroplastic-like isoform X2	1.14	1.03
PaTc_017600	PF03936	378	TPS-a			XP_022012803.1	alpha-copaene synthase-like	0.71	0.03
PaTc_021219	PF03936	492	TPS-e/f	0.54	1.00	XP_022018100.1	ent-kaur-16-ene synthase, chloroplastic-like	0.47	0.76
PaTc_025290	PF03936	418	TPS-a	-2.60	0.22	XP_021969440.1	(-)-germacrene D synthase-like	7.92	1.36
PaTc_025293	PF03936	214	TPS-a	-3.64	0.44	AMP42987.1	germacrene D	21.05	1.66
PaTc_025384	PF03936	389	TPS-g			XP_021992882.1	(3S,6E)-nerolidol synthase 1-like	0.47	0.35
PaTc_070261	PF03936	172	TPS-b	-2.59	0.70	XP_022000736.1	R-linalool synthase QH1, chloroplastic-like	0.69	0.11
PaTc_070262	PF03936	429	TPS-b	-2.49	0.06	XP_022000734.1	R-linalool synthase QH1, chloroplastic-like	3.16	0.55
**PaTc_072148**	**PF03936**	**516**	**TPS-b**	**-4.32**	**0.00**	**AMP42989.1**	**putative monoterpene synthase**	**28.84**	**1.42**

Table S6. Identification and expression analysis of selected carbohydrate metabolism genes in guayule

Gene Name	Contig	logFC	FDR	Ctrl_TMM	Drought_TMM	Accession No.	Annotation
Drought stress (this study)							
FEH1	PaTc_110755	3.53	0.01	9.94	120.49	OTG32010.1	fructan exohydrolase
FEH1	PaTc_110757	2.36	0.25	0.84	4.32	OTG32010.1	fructan exohydrolase
FEH1	PaTc_110759	0.25	1.00	1.87	2.29	OTG32010.1	fructan exohydrolase
FFT1	PaTc_016978	-4.93	0.02	2.31	0.06	CAA08811.1	fructan 1-fructosyltransferase
FFT1	PaTc_016980	-3.58	0.00	265.96	21.60	CAA08811.1	fructan 1-fructosyltransferase
SST1	PaTc_199271	-5.59	0.50	166.97	3.42	XP_021974022.1	sucrose-1-fructosyltransferase
SST1	PaTc_199269	-5.57	0.02	729.11	15.03	XP_021974022.1	sucrose-1-fructosyltransferase
FBA1	PaTc_033225	3.02	0.00	16.14	128.59	XP_022015802.1	fructose-bisphosphate aldolase 1, chloroplastic-like
FEH6	PaTc_004249	3.54	0.05	0.19	2.15	XP_022023405.1	fructan 6-exohydrolase-like
VTC2	PaTc_066454	2.26	0.01	9.05	44.16	XP_022039518.1	GDP-L-galactose phosphorylase 1-like
n.a	PaTc_068062	2.43	0.02	2.54	13.48	XP_022033606.1	beta-glucosidase 18-like isoform X1
n.a	PaTc_166809	-3.82	0.04	0.78	0.05	XP_022028361.1	glucose-1-phosphate adenylyltransferase large subunit 1-like isoform X1
RFS6	PaTc_066497	-2.04	0.02	7.79	1.89	XP_021990267.1	probable galactinol--sucrose galactosyltransferase 6

Gene Name	Contig	logFC	FDR
Cold stress (Stonebloom and Sheller, 2019)			
SST1	GFTW01094278.1	-1.94	0.00
SST1	GFTW01084468.1	-1.98	0.02
FFT1	GFTW01094277.1	-0.51	0.07
FFT1	GFTW01084466.1	-1.69	0.15
FEH1	GFTW01080337.1	1.07	0.45

Table S7. List of primers used for RT-qPCR analysis

Gene Name	Primer sequence (5' to 3')	Product size (bp)
3-Hydroxy-3-methylglutaryl coenzyme A synthase (HMGS)	Forward: GGTTCAGCCAGGAACTTTGG<br>Reverse: GGAAGCAAATGCAGCATATAAAGA	76
3-Hydroxy-3-methylglutaryl coenzyme A reductase 1 (HMGR1)	Forward: AGAAGAAGAAGAGGAAGAGGAG<br>Reverse: TCTTCGGTCACTGTTGTTTC	72
3-Hydroxy-3-methylglutaryl coenzyme A reductase 2 (HMGR2)	Forward: ATGGGCATTTCTGGAAACTATTG<br>Reverse: CTTCCCTCGCCCTTCTATCC	156
Farnesyl pyrophosphate synthase (FPPS)	Forward: TCAACGATCCTGCCTTCGA<br>Reverse: TCCAGGTACGTTGTAGTCAAGCA	77
cis-Prenyltransferase 3 (CPT3)	Forward: GCTTCTTTTTCGGGTCATTTCA<br>Reverse: TGCCAAGAATCCGGCTTTAT	130
cis-Prenyltransferase 3 binding protein (CBP)	Forward: GGCGGTCATCATGGAGAGA<br>Reverse: GATTGGCTACTGCACTATCATTGG	62
Allene oxide synthase 1 (AOS1)	Forward: CACGGTATTTCGAGCCAACA<br>Reverse: CGTCGAGTAGGACGATGACCTT	74
Small Rubber Particle Protein (SRPP)	Forward: GTGGCCAACACATTGTACGTAAA<br>Reverse: TTCTCAGCTACCGGCTCGTAGT	77
Eukaryotic translation initiation factor 4 alpha (eIF4a)	Forward: TTGAATGCCAGGCTTTGGTT<br>Reverse: GCCCGCATGACCTTCTCA	70
18S Ribosomal RNA (18S)	Forward: TACTATGGTGGTGACGGGTG<br>Reverse: ATTGTCACTACCTCCCCGTG	133
